# Assessment of rosacea symptom severity by genome-wide association study and expression analysis highlights immuno-inflammatory and skin pigmentation genes

**DOI:** 10.1093/hmg/ddy184

**Published:** 2018-05-16

**Authors:** Jennifer L Aponte, Mathias N Chiano, Laura M Yerges-Armstrong, David A Hinds, Chao Tian, Akanksha Gupta, Cong Guo, Dana J Fraser, Johannes M Freudenberg, Deepak K Rajpal, Margaret G Ehm, Dawn M Waterworth

**Affiliations:** 1 Genomic Medicine, PAREXEL International, Research Triangle Park, NC, USA; 2 Target Sciences, GlaxoSmithKline, Stevenage, UK; 3 Target Sciences, GlaxoSmithKline, Collegeville, PA, USA; 4 23andMe Inc., Mountain View, CA, USA; 5 Translational Science, Dermatology, GlaxoSmithKline, Research Triangle Park, NC, USA

## Abstract

Rosacea is a common, chronic skin disease of variable severity with limited treatment options. The cause of rosacea is unknown, but it is believed to be due to a combination of hereditary and environmental factors. Little is known about the genetics of the disease. We performed a genome-wide association study (GWAS) of rosacea symptom severity with data from 73 265 research participants of European ancestry from the 23andMe customer base. Seven loci had variants associated with rosacea at the genome-wide significance level (*P *<* *5 × 10^−8^). Further analyses highlighted likely gene regions or effector genes including *IRF4* (*P* = 1.5 × 10^−17^), a human leukocyte antigen (HLA) region flanked by *PSMB9* and *HLA-DMB* (*P* = 2.2 × 10^−15^), *HERC2-OCA2* (*P* = 4.2 × 10^−12^), *SLC45A2* (*P* = 1.7 × 10^−10^), *IL13* (*P* = 2.8 × 10^−9^), a region flanked by *NRXN3* and *DIO2* (*P* = 4.1 × 10^−9^), and a region flanked by *OVOL1*and *SNX32* (*P* = 1.2 × 10^−8^). All associations with rosacea were novel except for the HLA locus. Two of these loci (*HERC-OCA2* and *SLC45A2*) and another precedented variant (rs1805007 in melanocortin 1 receptor) with an association *P* value just below the significance threshold (*P* = 1.3 × 10^−7^) have been previously associated with skin phenotypes and pigmentation, two of these loci are linked to immuno-inflammation phenotypes (*IL13* and *PSMB9-HLA-DMA*) and one has been associated with both categories (*IRF4*). Genes within three loci (*PSMB9-HLA-DMA*, *HERC-OCA2* and *NRX3-DIO2*) were differentially expressed in a previously published clinical rosacea transcriptomics study that compared lesional to non-lesional samples. The identified loci provide specificity of inflammatory mechanisms in rosacea, and identify potential pathways for therapeutic intervention.

## Introduction

Over 16 million Americans are affected with rosacea, a chronic inflammatory skin disease with poorly understood etiology, genetics and pathophysiology (1,2). Studies have suggested that rosacea may occur three times more often in women than in men. Although women are more likely to experience symptoms on the cheeks and chin, men are more likely to have severe symptoms and are more commonly affected on the nose (3). Although the clinical presentation is highly variable, rosacea has been classified into four common subtypes: (i) erythematotelangiectatic rosacea (ETR) characterized by flushing and persistent redness on face; (ii) papulopustular rosacea (PPR) characterized by persistent facial redness with papules or pustules that come and go; (iii) phymatous rosacea (PhR) characterized by thickening skin, irregular nodularities and enlargement primarily of the nose and (iv) ocular rosacea where the eyes display a host of symptoms that may include irritation, dryness, stinging, light sensitivity and decreased visual acuity (1). Rosacea patients often experience progression from one subtype to another (4). The onset of rosacea typically occurs between the ages of 30 and 60 years as intermittent facial redness, progressing into visible blood vessels, bumps, papules, pustules, eye irritation and swelling of the nose if left untreated (5).

The exact pathogenesis of rosacea is unknown, but dysregulation of the innate and adaptive immune system, overgrowth of commensal skin organisms and abnormal neurovascular signaling may all have a role (1). Studies suggest that rosacea occurs in individuals with an inappropriate innate immune response to environmental triggers, such as stress and sun exposure, leading to inflammation and vascular abnormalities (6). A recent transcriptome-wide study of biopsies from rosacea patients and healthy volunteers suggested distinct differences between three rosacea subtypes (ocular rosacea not reported) and observed that all subtypes are characterized by Th1/Th17 polarized inflammation, and macrophage and mast cells infiltration (7). Despite progress in understanding pathopysiology of rosacea, work remains to understand the complex dysregulation of cells and mechanisms of disease response as well as genetic determinants of disease. Twin studies have demonstrated that genetics may contribute to rosacea incidence and severity (8), but only one genome-wide scan has been performed to date, also with 23andMe, Inc. research participants (9). The study identified an association between rosacea risk and a single nucleotide polymorphism (SNP) in the human leukocyte antigen (HLA) region located between *HLA-DRA* and *BTNL2*, which supported the concept of an inflammatory genetic component to rosacea. However, the study’s sample size was limited to 22 952 individuals, employed a binary disease definition (2618 cases/20 334 controls) and did not investigate rosacea symptom severity. The absence of animal models further emphasizes the need to conduct translational and genetics research to dissect molecular pathways that can be therapeutically targeted (10). We performed a genome-wide association study (GWAS) in a large cohort of 73 265 individuals of European ancestry who responded to a rosacea symptom severity questionnaire to increase power and gain insight into the molecular basis of rosacea symptom severity.

## Results

A GWAS was conducted in 73 265 individuals who self-reported about their rosacea symptoms and had greater than 97% European ancestry, as determined through an analysis of local ancestry. Rosacea symptom severity was evaluated using a composite 32-point score derived from survey questions about rosacea-related facial, nose and ocular symptoms analyzed as a continuous trait. Association analysis used a linear model adjusting for age, sex and the first five ancestry principal components. Table 1 summarizes the distribution of the rosacea symptom severity score for the participant group by age and gender. Although the number of men and women with symptoms of any severity are similar, the number of women reporting rosacea-like symptoms increases to three times that of men as the score increases. Higher scores were observed for women as compared with men and for the older age groups (>45 years) as compared with the 30–45 years age category.

**Table 1.** Severity score, gender and age characteristics of the GWAS participant group

Phenotype	Severity score group	Male	Female	Severity score group	Age (years) < 30	30–45	45–60	>60	Total
Rosacea symptom severity	[0–1]	21 809	12 584	[0–1]	10	9975	11 089	13 319	34 393
	(1–2]	4755	4676	(1–2]	1	2641	3158	3631	9431
	(2–3]	3386	4108	(2–3]	3	1980	2478	3033	7494
	(3–5]	3504	5620	(3–5]	2	2396	3141	3585	9124
	(5–8]	2152	4381	(5–8]	2	1732	2307	2492	6533
	(8–32]	1632	4658	(8–32]	8	1499	2498	2285	6290
		37 238	36 027		26	20 223	24 671	28 345	73 265

The online questionnaire was targeted to participants who self-reported as aged 30 years or older; hence, the majority of participants included in this analyses are >30 years of age. Rosacea symptom severity burden scores were derived using research participant data as of November 2014.

**Figure 1.**
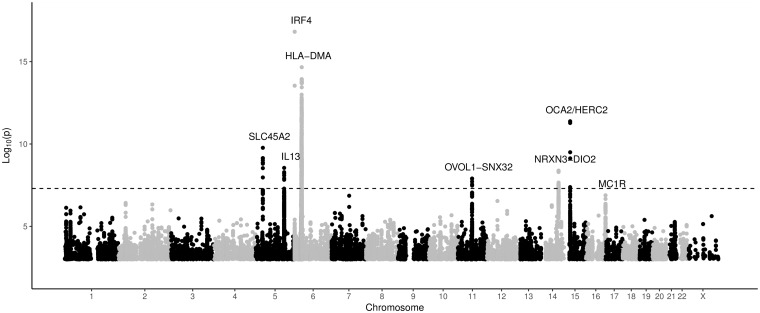
Manhattan plot of single-nucleotide polymorphisms in the rosacea GWAS group. The Manhattan plot depicts the distribution of association test results versus genomic position, with chromosomes 1 to 22, and X arranged along the *X* axis. The *Y* axis represents –log10 (*P* values). The *P* value threshold for declaring statistical significance at the GWAS level, *P* = 5 × 10^−8^, is indicated by the gray line. Loci with *P* < 5 × 10^−8^ are labeled with the name of the nearest gene. The *MC1R* gene association is annotated on the plot as well.

The results of the GWAS are shown in Figure 1. Variation within seven genomic regions showed significant association with rosacea symptom severity at the genome-wide level (*P *<* *5* *×* *10^−8^). Patterns of association in these genomic regions are illustrated using regional plots (Supplementary Material, Fig. S1). Summary statistics for all variants with suggestive associations (*P < *5 × 10^−5^) to rosacea symptom severity are listed in Supplementary Material, Table S1. Although the genomic control value was large (*λ*_GC_ of 1.0864), linkage disequilibrium (LD) score regression analysis had an intercept of 1.0099 suggesting that polygenicity accounts for the majority of the increase in the mean *χ*^2^ statistic. We analyzed the associated loci with the Probabilistic Identification of Causal SNPs (PICS) method which uses LD and the pattern of association in order to determine the most likely causal variant or variants (referred to as the PICS set, Supplementary Material, Table S2) (11). We calculated the PICS probability assuming a European reference ancestry for each associated locus and identified the most likely causal SNP or SNPs (i.e. the PICS set) using a ≥90% probability threshold. Chromatin regulatory marks in blood and immune cells were evaluated to identify possible regulatory variants in each region (Supplementary Material, Figs S2–S7). To provide support for proposed effector genes within associated loci, variants within these regions were further evaluated to determine *cis* eQTLs using publicly available data in GTEx (12) (Supplementary Material, Table S3). Summary information for the most significant SNP in each region, including the most likely ‘effector’ gene for each locus, as well as information on previous reports implicating these variants can be found in Table 2.

**Table 2.** Characteristics of the most significant GWAS SNPs associated with rosacea symptom severity score

rsID	Chromosome position	Allele	MAF	Gene context	Possible effector gene(s)	*P* value	Effect *β* (95% CI)	Gene description	Prior published associations with index SNP or SNP(s) within 500 kb and moderate LD (*r*^2^ > 0.5)
rs12203592	6p25.3: 396321	C/T	0.174	*[IRF4]*	*IRF4*	1.5 × 10^−17^	0.221 (0.169, 0.273)	Interferon regulatory factor 4	Hair color (14,35,36), eye color (14,35), skin color (14), freckling (35), non-melanoma skin cancer (36), sunburns/tanning (14,36)
rs57390839	6p21.32: 32900383	TT/–	0.07	*PSMB9–[]- HLA-DMB*	*HLA-DMA, HLA-DMB*	2.2 × 10^−15^	–0.303 (–0.380, –0.227)	HLA class II histocompatibility antigen	Autoimmune and inflammatory disease (21)
rs1129038	15q13.1: 28356859	C/T	0.266	*[HERC2]*	*OCA2, HERC2*	4.2 × 10^−12^	0.157 (0.111, 0.202)	Probable E3 ubiquitin-protein ligase	Generalized vitiligo (40), tanning (36), eye color (35,36,57–61), hair color (14,35,36,57)
rs16891982	5p13.2: 33951693	C/G	0.042	*[SLC45A2] (MATP)*	*SLC45A2*	1.7 × 10^−10^	0.317 (0.218, 0.202)	Solute carrier family 45 member 2	Hair color (14,35), eye color (35), skin pigmentation (64), tanning (65), malignant melanoma (66)
rs847	5q31.1: 131996669	C/T	0.2	*[IL13]*	*IL13*	2.8 × 10^−9^	–0.143 (–0.191, –0.095)	Interleukin 13	Asthma (23,24), atopic dermatitis (25,26), psoriasis (28), self-reported allergy (27), Hodgkin’s lymphoma (62), IgE levels (63)
rs149851565	14q31.1: 80509104	C/A	0.116	*NRXN3—[]—DIO2*		4.1 × 10^−9^	0.175 (0.115, 0.235)		No previous associations
rs77779142	11q13.1: 65599656	C/T	0.159	*OVOL1–[]-SNX32*		1.2 × 10^−8^	0.150 (0.097, 0.203)		IBD (18)
rs1805007	16q24.3: 89986117	C/T	0.074	*TCF25-[]-TUBB3*	*MC1R*	1.3 × 10^−7^	0.189 (0.117, 0.261)	Melanocortin 1 receptor	Hair color (14,36,57), skin sensitivity to sun (57), tanning (36), freckling (35,57), basal cell carcinoma (67), non-melanoma skin cancer (36), melanoma (66,68,69), homocysteine levels (70)

The table of index or most significant SNPs in each associated locus. Regions were defined by identifying SNPs with *P* < 1 × 10^−5^, then grouping these into intervals separated by gaps of at least 250 kb, and choosing the SNP with smallest *p* within each interval. Mapping and gene context was based on NCBI Build 37, and the gene context for the most likely genes were derived using the HG19 release of the UCSC Known Genes tables. The gene context field has the following interpretations: Gene1, Gene2: The SNP is contained within the transcripts of the specified gene(s); Gene1−[]−Gene2: The SNP is flanked by Gene1 and Gene2. Dashes indicate distance: ‘−’= <10 kb, ‘−−’= <100 kb, ‘−−−’= <1000 kb. The two SNP alleles are in order of major/minor allele. Putative effector genes at each locus were identified by first defining the credible set of SNPs via PICS, and then annotating variants and genes within each credible set using functional fine mapping, differential expression analysis, and prior evidence of association.

Of the seven loci, two had previously been genetically associated with skin phenotypes and pigmentation (*SLC45A2* and *HERC2-OCA2*), two with immuno-inflammation phenotypes (*IL13* and *HLA-DMA/B*) and one gene (*IRF4*) was previously associated with both phenotypic categories. Two loci were intergenic (*NRX3-DIO2* and *OVOL1*-*SNX32*) and did not contain any obvious candidate genes for rosacea. In addition to these loci, another variant with a *P* value just below the significance threshold, rs1805007 in melanocortin 1 receptor (*MC1R*; *P *<* *1.3 × 10^−7^), has been previously genetically associated with skin phenotypes and pigmentation.

**Figure 2.**
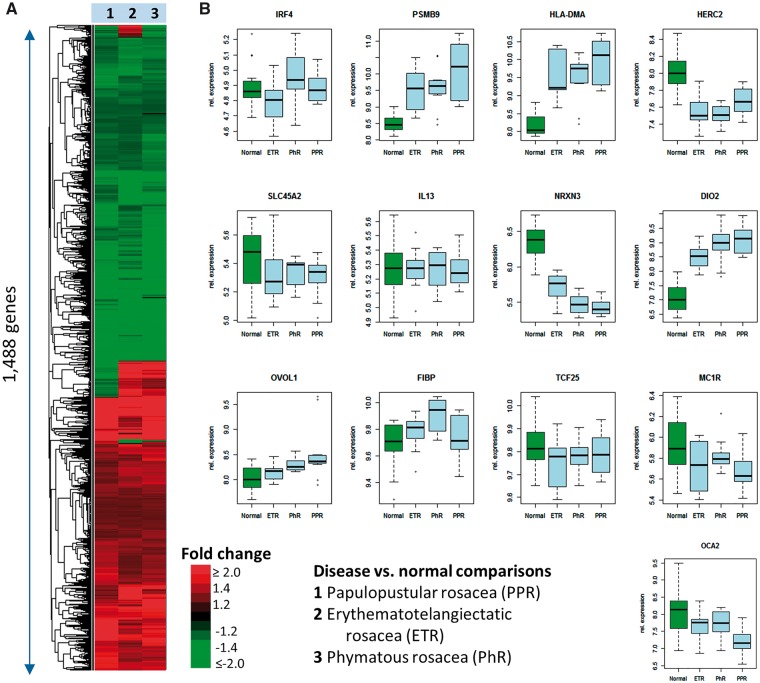
Reanalysis of previously published mRNA expression in three rosacea subtypes (7). Samples from patients with PPR, ETR and PhR were compared with samples from healthy volunteers. The heatmap and hierarchical clustering of fold changes comparing rosacea samples against healthy signatures for each subtype shows that the three subtypes were highly similar with erythematotelangiectatic and PhR appearing more closely related than PPR (**A**). Differential expression analysis of selected genes showed that five genes in the eight gene regions that were associated with rosacea were differentially regulated in rosacea gene expression data (*PSMB9*_*HLA-DMA*, *HERC2* and *DIO2_NRX3*) (**B**).

A recently published microarray-based mRNA expression dataset (7) was reanalyzed to evaluate the genes and molecular pathways underlying rosacea and its subtypes that are dysregulated on the transcriptional level by comparing PPR, ETR and PhR samples to samples derived from healthy volunteers. Overall, transcriptional signatures for the three subtypes were highly similar (Fig. 2A). Differential expression analysis of the seven loci with *P* values less than 5.0 × 10^−8^ plus the *MCR1* locus showed that genes (including *HLA-DMA*, *PSMB9*, *HERC2*, *OCA2*, *NRX3* and *DIO2*) in three of the seven gene regions (*PSMB9-HLA-DMA*, *HERC2-OCA2* and *NRX3-DIO2)* were differentially regulated in rosacea gene expression data (Fig. 2B).

Our results show that the index SNP rs12203592 in the intronic region of the interferon regulatory factor 4 (*IRF4*) gene was strongly associated with rosacea symptom severity; the T allele is significantly associated with increased rosacea symptom severity (*P *=* *1.5 × 10^−17^). The associated variant is located within a melanocyte-specific enhancer (13) and is significantly associated with pigmentation traits in previously conducted GWAS (14). The rs12203592 variant is the only variant identified in the PICS set and resides in a gene regulatory element marked by active histone marks and open chromatin that is present in both skin and blood cell types (Supplementary Material, Fig. S2). Further, this variant is significantly associated with higher *IRF4* gene expression in transformed lymphocytes and whole blood in GTEx (12). Taken together these results indicate that rs12203592 may be impacting rosacea symptom severity through increased *IRF4* expression.

The second locus associated with rosacea symptom severity maps to the class II region of the major histocompatibility complex (MHC) at 6q21. The most significant SNP, rs57390839 (*P *=* *2.2 × 10^−15^), maps between genes *PSMB9* (proteasome subunit beta 9) and *HLA-DMB* (HLA class II histocompatibility antigen, DM beta chain). The variant represents a 2 bp deletion with a possible protective effect and maps most closely to *HLA-DMB* (40 kb upstream) and *HLA-DMA* (34 kb downstream). The rs57390839 variant has the highest likelihood of being related to rosacea on the basis of PICS (PICS probability = 1.0). There was no functional chromatin data available for this locus in existing databases. Multiple genes within this region also showed differential expression in the rosacea gene expression data, making it difficult to pin down the causal effector gene.

The third locus associated with rosacea symptom severity was at 15q13 (rs1129038, *P* = 4.2 × 10^−12^). This SNP is located in the 3′UTR of the *HERC2* (probable E3 ubiquitin-protein ligase) gene exon 93 and upstream of *OCA2* (probable E3 ubiquitin-protein ligase) and is part of a haplotype block that predicts eye color. Our results show that the minor allele (T) is significantly associated with increased rosacea symptom severity. Variant rs12913832, also significantly associated in our study, *P *=* *5.3 × 10^−12^, is located in an intron in *HERC2* affecting the expression of *OCA2* in the human iris, and is common to nearly all people with blue eyes (15). Two markers, rs1129038 and rs12913832, are in the PICS set and both overlap enhancers in skin cells. Although rs12913832 has a significant eQTL for *HERC2* in whole blood (GTEX, *P *=* *1.7 × 10^−6^), this may be a spurious or coincidental finding as the top eQTL for *HERC2* in whole blood is the different, nearby SNP rs8023410 (GTEX, eQTL *P *=* *1.7 × 10^−31^), which was only nominally associated with rosacea symptom severity (*P *=* *8.41 × 10^−6^). On the other hand, rs12913832 resides in a putative enhancer in foreskin melanocytes (Supplementary Material, Fig. S3) and has a higher PICS probability (0.66). Both *OCA2* and *HERC2* are significantly down-regulated in the rosacea gene expression data making it difficult to distinguish between *OCA2* and *HERC2* as the likely effector gene.

The fourth SNP associated with rosacea symptom severity (rs16891982, *P *=* *1.7 × 10^−10^) at 5p13 is in the solute carrier family 45 member 2 gene (*SLC45A2*), which encodes a transporter protein that mediates melanin synthesis with the G allele associated with increased symptom severity. Evaluation of the PICS data and the LD pattern across the region identified six SNPs likely to have a causal effect on rosacea. Of these, rs16891982 has the highest PICS probability (0.88) and is a missense variant (Leu374Phe) predicted to be functional (16). A gene regulatory mechanism is unlikely, because of a lack of overlap with active chromatin marks (Supplementary Material, Fig. S4). *SLC45A2* is already known to play a role in pigmentation phenotypes suggesting that it is the likely effector gene.

The most significant variant observed within the fifth locus is an intronic SNP (rs847) within the interleukin 13 (*IL13*) gene, with the minor allele (T) strongly associated with less severe forms of rosacea (*P *=* *2.8 × 10^−9^). This variant is in strong LD (*r*^2^ > 0.96) with a missense SNP, rs20541 (Gln144Arg), that is also associated with rosacea symptom severity (*P *=* *8.1 × 10^−9^). IL13 is a cytokine derived from helper T-cell lymphocytes that inhibits mononuclear cell inflammation (17). Five SNPs were identified in the PICS set (rs847, rs848, rs1295685, rs20541 and rs1295686). The SNPs rs1295686 and rs20541 reside within a putative enhancer in B-lymphocytes (Supplementary Material, Fig. S5) and all, except the rs848 variant, were associated with IL13 expression in testis in GTEX (*P *<* *7 × 10^−6^). Given the patterns of association and LD across the locus, this association signal is likely modulating IL13 expression and inflammation.

At the next locus, the strongest association was with an intergenic variant SNP located in the *NRXN3-DIO2* genomic region at 14q31 (rs149851565, *P *=* *4.1 × 10^−9^), with the minor allele (A) associated with increased rosacea symptom severity. There are no previous associations reported for this SNP and no significant eQTLs are reported in this region. Determining the effector gene for this region is further complicated by the fact that the associated variants are in high LD with 53 other potentially causal variants and by the lack of robust overlap with regulatory markers in relevant tissues (Supplementary Material, Fig. S6).

For the final locus, the most significant association is with an intergenic variant located between *OVOL1-SNX32* at 11q13 (rs77779142, *P *=* *1.2 × 10^−8^), with the T allele associated with more severe forms of rosacea. No previous associations are reported for this SNP; however, this variant is in high LD (*r*^2^ = 0.94) with rs2231884 that has previously been associated with inflammatory bowel disease (IBD) (18). Within the locus, 32 SNPs that may be causally related to rosacea overlap enhancer and promoter sites in skin and blood. The SNP rs17854357 is located in a blood and skin cell promoter of *SNX32*. The SNP rs11227332 resides within a regulatory element present in both tissue types near *CFL1*. SNPs rs12225345 and rs658524 are located within a blood cell type-specific promoter element. Lastly, there is a cluster of variants in the promoter of *FIBP* (Supplementary Material, Fig. S7). The plethora of regulatory mechanisms at the locus makes it difficult to pinpoint a causal mechanism. In GTEx, there are multiple significant (*P *<* *1 × 10^−11^) eQTLs in multiple tissues including skin in GTEx for genes in this region (*SNX32*, *CTSW* and *FIBP*). However, only the eQTL results for *SNX32* were concordant with the most significant eQTL results for that gene making the *CTSW* and *FIBP* results more likely coincidental or spurious. In addition, *SNX32*, *FIBP* and *CTSW* were not differentially regulated in the rosacea gene expression data. This evidence, collectively, does not point to a clear effector gene in this locus.

In addition to the seven loci described, a non-synonymous deleterious missense variant (Arg151Cys) mapping to the *MC1R* gene was associated with rosacea symptom severity and the (T) allele associated with more severe forms of the disease, but did not meet the threshold for genome-wide significance (*P *=* *1.3 × 10^−7^). The pattern of association and LD across this region suggests that this variant, as well as 23 other variants within this locus could be causally related to rosacea. *MC1R* plays an important role in normal pigmentation (see Table 2). The receptor is primarily located on the surface of melanocytes, specialized cells that produce melanin. There are numerous eQTL associations in GTEx in multiple tissues including brain but no significant signals were identified for skin.

Given the overlap observed with immune and pigmentation loci, we evaluated the genetic correlation between rosacea symptom severity and relevant conditions where GWAS summary statistics were available using LD score regression. Unfortunately, pigmentation GWAS were not available, but we compared the association results with 10 other GWAS for immune conditions. We observed significant (*P *<* *0.05/10 = 0.005) positive genetic correlations with IBD (*r_G_* = 0.228; *SE_rG_* = 0.069; *P *=* *0.001) and ulcerative colitis (UC) (*r_G_* = 0.217; *SE_rG_* = 0.074; *P *=* *0.003), Table 3 summarizes results from the analysis. Several large epidemiological studies have shown correlations between these conditions, with a UK study indicating an increased risk for rosacea in patients with a history of UC (19) and the Nurse’s Health Study in the US demonstrating an increased risk of Crohn’s disease in women with a history of rosacea (20). Therefore, evidence exists connecting these two conditions, but more investigation is required to understand causality.

**Table 3.** Genetic correlation between rosacea symptom severity score and other immune mediated diseases

Trait	r_G_	SE_r_G_	*P*
Eczema (71)	–0.268	0.122	0.028
Crohn’s disease (72)	0.187	0.07	0.008
IBD (72)	0.228	0.069	0.001
UC (72)	0.217	0.074	0.003
Asthma (73)	–0.094	0.116	0.417
Rheumatoid arthritis (74)	–0.031	0.068	0.649
Multiple sclerosis (75)	0.109	0.158	0.492
Systemic lupus erythematosus (76)	0.132	0.083	0.113
Primary biliary cirrhosis (77)	0.199	0.094	0.033
Celiac disease	0.2409	0.107	0.024
Primary sclerosing cholangitis	–0.0815	0.122	0.5039

rG refers to the genetic correlation between rosacea symptom severity and the listed traits, SEr_G_ is the standard error of the genetic correlation and *P* is the *P* value of the genetic correlation.

## Discussion

In this study of rosacea symptom severity, we leveraged a large database of research participants’ self-reported data with rosacea-related symptom information and identified novel susceptibility loci. This information coupled with gene expression analyses highlighted gene regions previously implicated in skin pigmentation (*HERC2-OCA2*, *SLC45A2* and *MC1R*), inflammation (*IL13*, *HLA* and potentially *OVOL1*-*SNX32*) or both (*IRF4*), supporting the hypothesis that both skin type and inflammatory mechanisms play a critical role in rosacea pathophysiology. LD score regression analysis also demonstrated significant correlation with the genetic associations in the immune conditions, IBD and UC. Together with the inflammatory mechanisms identified in the published transcriptomics study (7), this study further highlights the importance of understanding immune-mediated mechanisms and to explore them further for therapeutic resolution. This study comprises more than 73 000 participants and provides insight beyond the HLA region, previously associated with rosacea risk (9). Supplementary Material, Table S4 summarizes the case and control status for participants included in the present study and the primary cohort published in Chang et al. (9) illustrating that participants with higher scores are more likely to be diagnosed with rosacea versus those with lower scores and that the symptom score captures information beyond a traditional doctor diagnosis.

Analysis of these data provided insight into the value of self-reported data. The phenotype analyzed in this study included a burden score that has not been previously studied using a genome scan approach. Even though this is not a clinically validated score, the increased yield of significant results beyond the simple case–control definition suggests that this score is meaningful in disease pathogenesis terms and supports the idea of a continuum of disease symptoms across the subtypes. Although the loci identified in this study have not been replicated, the size of the study, the strength of statistical associations and the likely functional impacts of the effector genes in these regions provide compelling evidence for prioritizing these loci for further in-depth investigations.

The associations of rosacea with the *IL13*, *IRF4* and *HLA* gene regions are consistent with the known inflammatory component of the disease. The *HLA-DMA/B* genes that map most closely to our associated variant are part of the MHC class II, expressed in antigen presenting cells. Many associations with autoimmune conditions have been shown to be specific to class II alleles (21) including susceptibility to rheumatoid arthritis, multiple sclerosis and celiac disease. For rosacea, it has also been suggested that HLA associations may help explain the connection of various microbes with rosacea (22)*.* The *HLA* gene locus identified here for rosacea symptom severity overlaps with a region previously associated with rosacea risk (9). Although our study provides greater power to precisely dissect contributions of MHC variants to rosacea symptoms, accurate mapping of disease loci and detailed interpretation of MHC-disease associations is still problematic because of the density of MHC genes, the strong LD between genes and the effects of multiple HLA loci (21).

IL-13 is a cytokine secreted by many cell types, but especially T helper type 2 (Th2) cells (17), and is a mediator of allergic inflammation and disease. Our associated SNPs in the region, rs1295686 and rs20541, have been previously implicated in autoimmune diseases (rs1295686 with asthma (23,24) and atopic dermatitis (25,26), and rs20541 with self-reported allergy (27) and psoriasis (28)). However, not all these associations are in the same direction, for example the minor allele of rs20541 confers risk of atopic dermatitis, but is protective for rosacea and psoriasis. Recent studies also report positive findings on the efficacy of new therapies targeting cytokines interleukin IL-13 and IL-4 in asthma and atopic dermatitis (29,30).

IRF4, a T cell receptor-induced transcription factor, functions in partnership with multiple molecular partners to modulate adaptive immune response (31). *IRF4* plays a role in the initiation and progression of autoimmune diseases (32) such as celiac disease (33) and is strongly associated with human pigmentation traits (34). The *IRF4* variant described here affects skin color by modulating enhancer-mediated transcriptional regulation (13) and has been previously associated with hair color (14,35,36) eye color (14,35), skin color (14), freckling (35), non-melanoma skin cancer (36) and sunburn/tanning (14,36). Additionally, *IRF4* plays a critical role in the fate of T-cells as it promotes differentiation of naïve CD4(+) T cells into various cell types such as T helper 2 (Th2), Th9, Th17 or T follicular helper (Tfh) cells (37). These results support the possibility of *IRF4* playing a dual role in influencing skin susceptibility to rosacea as well as a potentially mediating inflammation in the development of rosacea. Collectively, the *HLA*, *IL13* and *IRF4* findings highlight the critical role of immune modulation and rosacea disease pathology.

This study in individuals of 97% European ancestry has identified significant associations with rosacea genes previously implicated in skin pigmentation (*HERC2-OCA2*, *SLC45A2*, *IRF4* and *MC1R*). Skin pigmentation is primarily due to the function of melanocytes and cutaneous homeostatic mechanisms involving multiple cell types interacting with melanocytes. Melanosomes, components of melanocytes, synthesize two types of melanins: eumelanin (black-brown) and pheomelanin (red/yellow). The type and quality of melanin produced, and the cutaneous cell interactions determine the skin color of an individual. *MC1R* regulates the quality and quantity of melanin production and minor vitiligo autoantigen, and upon activation switches to eumelanin production (38). *MC1R* agonism along with ultraviolet B exposure assisting repigmentation has been reported (39). Associations between genetic variation in *MC1R* and malignant melanoma, vitiligo (a disease characterized by depigmented skin and hair resulting from autoimmune destruction of epidermal melanocytes) (40) and pigmentation traits have been reported (Table 2). Reduced production of melanin due to genetic mutations in the protein encoded by *SLC45A2* results in oculocutaneous albinism type IV (*OCA4*), an autosomal recessive inherited disorder with reduced melanin pigmentation in skin, hair and eyes (41). *SLC45A2* is also genetically associated with a variety of pigmentation phenotypes (Table 2). Another noteworthy association is with variation within the *HERC2-OCA2* locus, in particular a variant in an evolutionally conserved region of *HERC2* that downregulates expression of OCA2. *OCA2* is causal for oculocutaneous albinism type 2, encodes a melanosomal membrane transporter and has a major role in determining skin, hair and eye color (Table 2)*. HERC2* has also been associated with human pigmentation traits, and may have multiple roles in genomic stability (42). Further investigation is necessary to determine the mechanistic link between the gene variants identified in pigmentation genes and the rosacea phenotype.

This large study of rosacea symptom severity represents the first genome wide study of this phenotype. Together, these loci provide genetic insight into underlying disease processes in rosacea as a combination of skin type and susceptibility to inflammation. Further work will be needed to identify and replicate causal variants within associated loci and to assess functional variants identified as possible therapeutic targets.

## Materials and Methods

### Study design and 23andMe cohort

GWAS participants were customers of the genetics company 23andMe, Inc. (Mountain View, CA) who consented to research and answered surveys online according to the 23andMe human subjects protocol, which was reviewed and approved by Ethical & Independent Review Services, a private institutional review board (http://www.eandireview.com).

Responses to eight questions related to rosacea symptoms were added to generate a total symptom severity score, range 0–32 (Table 1). Participants responded to the following questions with a 0 (None), 1 (Slight), 2 (Mild), 3, (Moderate) or 4 (Severe) rating: ‘Do you have persistent redness or rosy appearance of the face?’, ‘Do you have tiny, visible blood vessels on the surface of your facial skin?’, ‘Do you have papules (small red bumps, sometimes filled with white pus) on the face?’, ‘Do you have a bulbous or cobbled appearance to your nose, cheeks, or chin?’, ‘Do you have frequent or prolonged flushing of the face, lasting up to several hours (can be triggered by eating hot or spicy foods, sun exposure, or temperature changes)?’, ‘Do you have burning or stinging of your face?’, ‘Do you have swelling of your face (facial edema)?’ and ‘Do you have a recurrent gritty feeling in your eyes or dry eyes?’.

The rosacea risk phenotype used in the GWAS previously reported (9) was defined on the basis of participant’s response to whether a healthcare professional had ever diagnosed them with rosacea. Participants who answered ‘yes’ were defined as ‘cases’ (*n* = 2618), and those who answered ‘no’ (*n* = 20 334) were defined as ‘controls.’ The symptom severity score in our study was derived at a later point in time and included individuals who responded to a survey related to rosacea symptoms independent of a diagnosis of rosacea and therefore included more subjects (73 265). Almost all participants that were studied as part of the GWAS of rosacea risk were included in our analysis but the variable under study in this case is a quantitative trait on the basis of rosacea-related symptoms ranging from ‘0’ (no rosacea symptoms) to ‘32’ (severe rosacea symptoms). Many patients who reported a diagnosis of rosacea in Chang et al. (9) also reported very mild symptomatology (Supplementary Material, Table S1). Moreover, many more patients with moderate to severe symptoms (score 5–32) were added in this analysis (*n* = 9389). Therefore, this sample set, the phenotype and these results are distinct to those described in the earlier paper. A cross-tabulation comparison of the cohorts across the two studies can be found in Supplementary Material, Table S1.

### Genotyping and quality control

Samples were genotyped on one of four Illumina genotyping platforms. The V1 and V2 platforms were variants of the Illumina HumanHap550+ BeadChip, including about 25 000 custom SNPs selected by 23andMe, totaling about 560 000 SNPs. The V3 platform was based on the Illumina OmniExpress+ BeadChip, with custom content with a total of about 950 000 SNPs. The V4 platform was a fully custom array with additional coverage of lower-frequency coding variation, and about 570 000 SNPs. Participant genotype data were imputed against the September 2013 release of 1000 Genomes Phase1 reference haplotypes. The genotyping platforms, quality control procedures, ancestry determination and imputation protocols have been previously described (43).

### GWAS analysis

Participants were restricted to a set of individuals who have >97% European ancestry, determined through an analysis of local ancestry (44). A maximal set of unrelated individuals, as defined by sharing no more than 700 cM IBD, was chosen for each analysis using a segmental identity-by-descent (IBD) estimation algorithm (45).

We tested for genetic associations using linear regression assuming an additive model for allelic effects, adjusting for age, gender, ancestry differences by including the first five GWAS principal components and genotype batch effects by including indicators for genotype platforms in the model: rosacea_severity ∼ age + sex + pc.0 + pc.1 + pc.2 + pc.3 + pc.4 + v2_platform + v3_1_platform + v4_platform + genotype. To test associations between HLA allele dosages and rosacea symptom severity, linear regression was performed using the same set of covariates used in the SNP-based GWAS. Separate association tests were performed for each imputed allele. Q–Q plots of the association *P* values for the study are shown in Supplementary Material, Figure S8.

Significant association was declared at the GWAS level (*P < *5.0 × 10^−8^). LD score regression was applied to the summary statistics to distinguish between polygenicity and test statistic inflation as implemented in LD Hub (www.ldsc.broadinstitute.org). We report the LD score regression intercept (46). All signals were evaluated for imputation quality (avg. *r*^2^ < 0.5 or min. *r*^2^ < 0.3) and association evidence provided by adjacent variants or variants in LD with the primary associated marker(s) to ensure robustness of the association.

The results were compared with other GWAS for autoimmune traits using LD score regression (46). Genetic correlation between traits was considered to be significant when the *P* value was less than 0.05 divided by the number of traits evaluated (*P *<* *0.05/10 = 0.005).

### *In silico* functional assessment

We estimated the probability that an individual SNP is the causal variant using a haplotype structure-based method called PICS for each of the top signals (11). Specifically, we calculated the PICS probability assuming a European reference ancestry for each associated locus and identified the most likely causal SNP or SNPs (i.e. the PICS set) using a ≥90% probability threshold.

We then completed functional annotation of SNPs in the PICS set in rosacea associated loci.

We annotated coding variation as well as putative regulatory information using HaploReg (47) and used *cis* eQTL data available in V6 of GTEx (GTEx Consortium). Active chromatin marks within each locus, excluding the HLA locus due to lack of data, were visualized in the UCSC Genome Browser (48). Tracks containing active histone marks (H3K27ac, H3K9ac, H3K4me3), open chromatin marks (DNase) and transcription factor binding sites (TF ChIP-seq) from multiple cell lines were loaded from the ENCODE Project (49) and the Roadmap Epigenomics Project (50). Represented skin cell lines include dermal fibroblast (NHDF), fetal skin (FSK), fetal abdominal skin (FFSA) and forekin melanocytes (PFM). Represented immune cell lines include B-lymphocytes (GM12878), CD8 primary cells, CD8 memory cells, CD4 primary cells and CD4 memory cells. The *cis* eQTL assessment was further refined by evaluating if the rosacea associated variant was concordant with the top *cis* eQTL for the identified tissue gene pairs as this would increase confidence that the colocalization of the *cis* eQTL and disease associations were not coincidental.

### Gene expression analysis

We reanalyzed a recently published rosacea mRNA expression dataset (7). This dataset included samples derived from 29 participants which were grouped as follows: ETR—7 patients, PPR—6 patients, PhR—6 patients, healthy volunteers—10 participants. Patients were diagnosed on the basis of the classification system of the American Rosacea Society (7). Total RNA was extracted from biopsies taken from the participants’ nasolabial fold and hybridized in duplicate to Affymetrix U133 plus 2.0 arrays (7). Raw data files (CEL files) were downloaded from the Gene Expression Omnibus (http://www.ncbi.nlm.nih.gov/geo/; accession GSE65914) (7) and then preprocessed using the RMA (Robust Multi-chip Average) pipeline (51) in combination with the most current reannotated probe set definitions (52). To determine differential mRNA expression, a linear model was fit using an empirical Bayes methodology for more robust variance estimates (53,54). The false discovery rate (FDR) was computed as an adjusted *P* value (55) to account for multiple testing and a cut-off of 10% FDR, as well as an absolute fold change of 1.5 or greater was used to define differential expression. Clustering analysis was performed as previously described (56).

## Data availability statement

The full summary statistics will be made available through 23andMe to qualified researchers under an agreement with 23andMe that protects the privacy of the 23andMe participants. Interested investigators should email dataset-request@23andme.com for more information and to apply to access the data.

## Supplementary Material

ddy184_supplmaterials_clean_v3

ddy184_suppltable1_hmg_v2
