# Supplementary material for: Assessment of rosacea symptom severity by genome-wide association study and expression analysis highlights immuno-inflammatory and skin pigmentation genes
Source: Hum Mol Genet. 2018 May 16;27(15):2762–72. doi: 10.1093/hmg/ddy184 (PMC6822543; doi:10.1093/hmg/ddy184)

**SUPPLEMENTARY FIGURES AND TABLES**

**Supplementary Table 1.  List of variants that provide evidence for association with rosacea**

Variants coordinates are based on NCBI Build 37.

* The Alleles field represent the two SNP alleles, A/B, for each SNP with B being the effect allele.

** Gene or closest gene mappings were constructed using the HG19 release of the UCSC known genes tables, described here: Gene1, Gene2: The SNP is contained within the transcripts of the specified gene(s), Gene1−−[]−−−Gene2: The SNP is flanked by Gene1 and Gene2. Dashes indicate distance: ‘’ = <1kb, ‘−‘ = <10kb, ‘−−‘ = <100kb, ‘−−−‘ = <1000kb.

Gene1−−[]: SNP is flanked by Gene1 on the left, but there is no gene within 1000kb on the right. Similarly, []−−−Gene2, implies SNP is flanked by Gene2 on the right, but there is no gene within 1000kb on the left.

| **Supplementary Table 2. PICS set** | | | | |
| --- | --- | --- | --- | --- |
| Index SNP | Locus | SNPs in PICs Set | r^2^ with Index SNP | PIC Score |
| rs12203592 | 6p25.3 | rs12203592 | 1 | 0.9999 |
| rs57390839 | 6p21.32 | rs57390839 | 1 | 1 |
| rs1129038 | 15q13.1 | rs1129038 | 1 | 0.6567 |
|  |  | rs12913832 | 0.9872 | 0.3372 |
| rs16891982 | 5p13.2 | rs16891982 | 1 | 0.8826 |
|  |  | rs35407 | 0.7331 | 0.0193 |
|  |  | rs35395 | 0.7331 | 0.0193 |
|  |  | rs35389 | 0.7331 | 0.0193 |
|  |  | rs28777 | 0.7331 | 0.0193 |
|  |  | rs185146 | 0.7331 | 0.0193 |
| rs847 | 5q31.1 | rs847 | 1 | 0.2646 |
|  |  | rs848 | 1 | 0.2646 |
|  |  | rs1295685 | 1 | 0.2646 |
|  |  | rs20541 | 0.9702 | 0.1085 |
|  |  | rs1295686 | 0.9628 | 0.0969 |
| rs77779142 | 11q13.1 | rs77779142 | 1 | 0.2758 |
|  |  | rs111953392 | 0.9902 | 0.1704 |
|  |  | rs17854357 | 0.9708 | 0.1183 |
|  |  | rs2231884 | 0.8688 | 0.0375 |
|  |  | rs58568715 | 0.8593 | 0.0342 |
|  |  | rs12225345 | 0.8425 | 0.029 |
|  |  | rs201407003 | 0.8028 | 0.0198 |
|  |  | rs11227333 | 0.787 | 0.017 |
|  |  | rs11227332 | 0.7825 | 0.0162 |
|  |  | rs658524 | 0.7638 | 0.0135 |
|  |  | rs645900 | 0.7638 | 0.0135 |
|  |  | rs601863 | 0.7638 | 0.0135 |
|  |  | rs1204650 | 0.7638 | 0.0135 |
|  |  | rs1151519 | 0.7638 | 0.0135 |
|  |  | rs583887 | 0.757 | 0.0126 |
|  |  | rs694994 | 0.7544 | 0.0123 |
|  |  | rs693824 | 0.7544 | 0.0123 |
|  |  | rs687672 | 0.7544 | 0.0123 |
|  |  | rs677931 | 0.7544 | 0.0123 |
|  |  | rs658938 | 0.7544 | 0.0123 |
|  |  | rs568617 | 0.7544 | 0.0123 |
|  |  | rs10791830 | 0.752 | 0.012 |
|  |  | rs596002 | 0.7503 | 0.0118 |
|  |  | rs656980 | 0.7477 | 0.0115 |
|  |  | rs641018 | 0.7477 | 0.0115 |
|  |  | rs588114 | 0.7318 | 0.0098 |
|  |  | rs507062 | 0.7318 | 0.0098 |
|  |  | rs78028320 | 0.7096 | 0.0077 |
|  |  | rs148654891 | 0.7096 | 0.0077 |
|  |  | rs144367425 | 0.7096 | 0.0077 |
|  |  | rs12283874 | 0.7096 | 0.0077 |
|  |  | rs10896050 | 0.7096 | 0.0077 |
| rs149851565 | 14q31.1 | rs149851565 | 1 | 0.1867 |
|  |  | rs75861276 | 0.9557 | 0.0626 |
|  |  | rs11847878 | 0.946 | 0.0552 |
|  |  | rs78832399 | 0.9347 | 0.0481 |
|  |  | rs17110253 | 0.9011 | 0.0331 |
|  |  | rs10150048 | 0.8968 | 0.0316 |
|  |  | rs185676995 | 0.8909 | 0.0297 |
|  |  | rs141699251 | 0.8788 | 0.0262 |
|  |  | rs4641659 | 0.8784 | 0.0261 |
|  |  | rs190863203 | 0.8687 | 0.0236 |
|  |  | rs10139915 | 0.8519 | 0.0199 |
|  |  | rs55806367 | 0.8494 | 0.0194 |
|  |  | rs10135581 | 0.8322 | 0.0163 |
|  |  | rs57474872 | 0.8297 | 0.0159 |
|  |  | rs67861209 | 0.8239 | 0.015 |
|  |  | rs17110240 | 0.8239 | 0.015 |
|  |  | rs75596007 | 0.8186 | 0.0143 |
|  |  | rs4140793 | 0.8186 | 0.0143 |
|  |  | rs17110255 | 0.8186 | 0.0143 |
|  |  | rs77195799 | 0.8139 | 0.0136 |
|  |  | rs77931578 | 0.8076 | 0.0128 |
|  |  | rs1024833 | 0.8052 | 0.0124 |
|  |  | rs10146401 | 0.8052 | 0.0124 |
|  |  | rs77576209 | 0.7992 | 0.0117 |
|  |  | rs17110251 | 0.7992 | 0.0117 |
|  |  | rs10483941 | 0.7966 | 0.0114 |
|  |  | rs8007550 | 0.7909 | 0.0108 |
|  |  | rs73324375 | 0.7882 | 0.0105 |
|  |  | rs56788873 | 0.7882 | 0.0105 |
|  |  | rs56067456 | 0.7882 | 0.0105 |
|  |  | rs17110189 | 0.7882 | 0.0105 |
|  |  | rs72685544 | 0.7874 | 0.0104 |
|  |  | rs8005680 | 0.7856 | 0.0102 |
|  |  | rs56214504 | 0.7847 | 0.0101 |
|  |  | rs28785299 | 0.7799 | 0.0096 |
|  |  | rs55730008 | 0.7738 | 0.009 |
|  |  | rs7142988 | 0.7718 | 0.0088 |
|  |  | rs10148855 | 0.7401 | 0.0063 |
|  |  | rs77091675 | 0.7391 | 0.0063 |
|  |  | chr14:80451159 | 0.7375 | 0.0061 |
|  |  | rs79725441 | 0.7329 | 0.0058 |
|  |  | rs67269821 | 0.7329 | 0.0058 |
|  |  | rs4140792 | 0.7329 | 0.0058 |
|  |  | rs12323429 | 0.7329 | 0.0058 |
|  |  | rs11847539 | 0.7329 | 0.0058 |
|  |  | rs185651297 | 0.7308 | 0.0057 |
|  |  | rs9989176 | 0.7293 | 0.0056 |
|  |  | rs9323682 | 0.7293 | 0.0056 |
|  |  | rs55746950 | 0.7259 | 0.0054 |
|  |  | rs4140791 | 0.7222 | 0.0052 |
|  |  | rs1477104 | 0.7186 | 0.005 |
|  |  | chr14:80457126 | 0.7186 | 0.005 |
|  |  | rs10483942 | 0.7115 | 0.0046 |
| rs1805007 | 16q24.3 | rs1805007 | 1 | 0.6647 |
|  |  | rs45610233 | 0.6833 | 0.0198 |
|  |  | rs77770855 | 0.6697 | 0.0173 |
|  |  | rs56850194 | 0.6697 | 0.0173 |
|  |  | rs74800773 | 0.6617 | 0.016 |
|  |  | rs146972365 | 0.6617 | 0.016 |
|  |  | rs113891247 | 0.6566 | 0.0152 |
|  |  | rs113955373 | 0.6439 | 0.0133 |
|  |  | rs78800020 | 0.6388 | 0.0127 |
|  |  | rs76581091 | 0.6388 | 0.0127 |
|  |  | rs75319471 | 0.6388 | 0.0127 |
|  |  | rs74336735 | 0.6388 | 0.0127 |
|  |  | rs73283871 | 0.6388 | 0.0127 |
|  |  | rs73283869 | 0.6388 | 0.0127 |
|  |  | rs73283867 | 0.6388 | 0.0127 |
|  |  | rs59574756 | 0.6388 | 0.0127 |
|  |  | rs59038611 | 0.6388 | 0.0127 |
|  |  | rs77606435 | 0.6262 | 0.0111 |
|  |  | rs73283861 | 0.6262 | 0.0111 |
|  |  | rs73283859 | 0.6262 | 0.0111 |
|  |  | rs112460025 | 0.6262 | 0.0111 |
|  |  | rs10584116 | 0.6262 | 0.0111 |
|  |  | rs201156703 | 0.6023 | 0.0086 |
| For each index SNP the full set of variants in the PICs set using a 90% probability threshold are provided. For each provided variant, the relevant score and the correlation with the index SNP in Europeans. | | | | |

| **Supplementary Table 3. Significant eQTL** | | | | | | |
| --- | --- | --- | --- | --- | --- | --- |
| Locus | Credible SNP | Genecode ID | Gene Symbol | *p v*alue | Effect Size | Tissue |
| 6p25.3 | rs12203592 | ENSG00000137265.10 | IRF4 | 3.00E-07 | 0.63 | Cells - EBV-transformed lymphocytes |
|  | rs12203592 | ENSG00000137265.10 | IRF4 | 6.00E-07 | 0.26 | Whole Blood |
| 15q13.1 | rs12913832 | ENSG00000128731.11 | HERC2 | 0.0000017 | -0.3 | Whole Blood |
| 5q31.1 | rs847 | ENSG00000169194.5 | IL13 | 5.40E-09 | 0.32 | Testis |
|  | rs1295685 | ENSG00000169194.5 | IL13 | 5.20E-09 | 0.33 | Testis |
|  | rs20541 | ENSG00000169194.5 | IL13 | 0.0000012 | 0.26 | Testis |
|  | rs1295686 | ENSG00000169194.5 | IL13 | 0.000007 | 0.25 | Testis |
| 11q13.1 | rs17854357 | ENSG00000172803.13 | [SNX32](javascript:portalClient.eqtl.gotoGeneExpression('SNX32')) | 1.20E-74 | 1.2 | [Cells - Transformed fibroblasts](javascript:portalClient.eqtl.goTissuePage('Cells_Transformed_fibroblasts')) |
|  | rs17854357 | ENSG00000172803.13 | [SNX32](javascript:portalClient.eqtl.gotoGeneExpression('SNX32')) | 2.30E-40 | 1.1 | [Esophagus - Muscularis](javascript:portalClient.eqtl.goTissuePage('Esophagus_Muscularis')) |
|  | rs17854357 | ENSG00000172803.13 | [SNX32](javascript:portalClient.eqtl.gotoGeneExpression('SNX32')) | 4.70E-34 | 1.1 | [Skin - Sun Exposed (Lower leg)](javascript:portalClient.eqtl.goTissuePage('Skin_Sun_Exposed_Lower_leg')) |
|  | rs17854357 | ENSG00000172803.13 | [SNX32](javascript:portalClient.eqtl.gotoGeneExpression('SNX32')) | 1.60E-33 | 1.1 | [Adipose - Subcutaneous](javascript:portalClient.eqtl.goTissuePage('Adipose_Subcutaneous')) |
|  | rs17854357 | ENSG00000172803.13 | [SNX32](javascript:portalClient.eqtl.gotoGeneExpression('SNX32')) | 3.40E-31 | 1.1 | [Thyroid](javascript:portalClient.eqtl.goTissuePage('Thyroid')) |
|  | rs17854357 | ENSG00000172803.13 | [SNX32](javascript:portalClient.eqtl.gotoGeneExpression('SNX32')) | 1.60E-29 | 1.1 | [Esophagus - Mucosa](javascript:portalClient.eqtl.goTissuePage('Esophagus_Mucosa')) |
|  | rs17854357 | ENSG00000172803.13 | [SNX32](javascript:portalClient.eqtl.gotoGeneExpression('SNX32')) | 2.60E-29 | 0.98 | [Nerve - Tibial](javascript:portalClient.eqtl.goTissuePage('Nerve_Tibial')) |
|  | rs17854357 | ENSG00000172803.13 | [SNX32](javascript:portalClient.eqtl.gotoGeneExpression('SNX32')) | 5.70E-28 | 1.1 | [Skin - Not Sun Exposed (Suprapubic)](javascript:portalClient.eqtl.goTissuePage('Skin_Not_Sun_Exposed_Suprapubic')) |
|  | rs17854357 | ENSG00000172543.3 | [CTSW](javascript:portalClient.eqtl.gotoGeneExpression('CTSW')) | 1.80E-26 | -0.8 | [Thyroid](javascript:portalClient.eqtl.goTissuePage('Thyroid')) |
|  | rs17854357 | ENSG00000172543.3 | [CTSW](javascript:portalClient.eqtl.gotoGeneExpression('CTSW')) | 6.90E-26 | -0.93 | [Esophagus - Mucosa](javascript:portalClient.eqtl.goTissuePage('Esophagus_Mucosa')) |
|  | rs17854357 | ENSG00000172803.13 | [SNX32](javascript:portalClient.eqtl.gotoGeneExpression('SNX32')) | 1.70E-25 | 0.96 | [Colon - Transverse](javascript:portalClient.eqtl.goTissuePage('Colon_Transverse')) |
|  | rs17854357 | ENSG00000172803.13 | [SNX32](javascript:portalClient.eqtl.gotoGeneExpression('SNX32')) | 2.00E-24 | 1.1 | [Artery - Aorta](javascript:portalClient.eqtl.goTissuePage('Artery_Aorta')) |
|  | rs17854357 | ENSG00000172803.13 | [SNX32](javascript:portalClient.eqtl.gotoGeneExpression('SNX32')) | 7.80E-23 | 1.1 | [Adipose - Visceral (Omentum)](javascript:portalClient.eqtl.goTissuePage('Adipose_Visceral_Omentum')) |
|  | rs17854357 | ENSG00000172803.13 | [SNX32](javascript:portalClient.eqtl.gotoGeneExpression('SNX32')) | 9.00E-23 | 0.87 | [Stomach](javascript:portalClient.eqtl.goTissuePage('Stomach')) |
|  | rs17854357 | ENSG00000172803.13 | [SNX32](javascript:portalClient.eqtl.gotoGeneExpression('SNX32')) | 1.40E-21 | 0.96 | [Lung](javascript:portalClient.eqtl.goTissuePage('Lung')) |
|  | rs17854357 | ENSG00000172543.3 | [CTSW](javascript:portalClient.eqtl.gotoGeneExpression('CTSW')) | 3.60E-21 | -0.91 | [Colon - Transverse](javascript:portalClient.eqtl.goTissuePage('Colon_Transverse')) |
|  | rs17854357 | ENSG00000172803.13 | [SNX32](javascript:portalClient.eqtl.gotoGeneExpression('SNX32')) | 2.10E-20 | 0.87 | [Artery - Tibial](javascript:portalClient.eqtl.goTissuePage('Artery_Tibial')) |
|  | rs17854357 | ENSG00000172803.13 | [SNX32](javascript:portalClient.eqtl.gotoGeneExpression('SNX32')) | 2.70E-19 | 1.4 | [Heart - Atrial Appendage](javascript:portalClient.eqtl.goTissuePage('Heart_Atrial_Appendage')) |
|  | rs17854357 | ENSG00000172803.13 | [SNX32](javascript:portalClient.eqtl.gotoGeneExpression('SNX32')) | 1.30E-18 | 1.1 | [Colon - Sigmoid](javascript:portalClient.eqtl.goTissuePage('Colon_Sigmoid')) |
|  | rs17854357 | ENSG00000172803.13 | [SNX32](javascript:portalClient.eqtl.gotoGeneExpression('SNX32')) | 1.70E-18 | 1.2 | [Brain - Cerebellum](javascript:portalClient.eqtl.goTissuePage('Brain_Cerebellum')) |
|  | rs17854357 | ENSG00000172803.13 | [SNX32](javascript:portalClient.eqtl.gotoGeneExpression('SNX32')) | 1.50E-17 | 1.1 | [Brain - Cortex](javascript:portalClient.eqtl.goTissuePage('Brain_Cortex')) |
|  | rs17854357 | ENSG00000172803.13 | [SNX32](javascript:portalClient.eqtl.gotoGeneExpression('SNX32')) | 4.20E-17 | 0.9 | [Breast - Mammary Tissue](javascript:portalClient.eqtl.goTissuePage('Breast_Mammary_Tissue')) |
|  | rs17854357 | ENSG00000172543.3 | [CTSW](javascript:portalClient.eqtl.gotoGeneExpression('CTSW')) | 1.30E-16 | -0.76 | [Nerve - Tibial](javascript:portalClient.eqtl.goTissuePage('Nerve_Tibial')) |
|  | rs17854357 | ENSG00000172803.13 | [SNX32](javascript:portalClient.eqtl.gotoGeneExpression('SNX32')) | 2.10E-16 | 1.3 | [Brain - Cerebellar Hemisphere](javascript:portalClient.eqtl.goTissuePage('Brain_Cerebellar_Hemisphere')) |
|  | rs17854357 | ENSG00000172543.3 | [CTSW](javascript:portalClient.eqtl.gotoGeneExpression('CTSW')) | 3.70E-16 | -0.85 | [Esophagus - Muscularis](javascript:portalClient.eqtl.goTissuePage('Esophagus_Muscularis')) |
|  | rs17854357 | ENSG00000172543.3 | [CTSW](javascript:portalClient.eqtl.gotoGeneExpression('CTSW')) | 4.20E-16 | -0.65 | [Skin - Sun Exposed (Lower leg)](javascript:portalClient.eqtl.goTissuePage('Skin_Sun_Exposed_Lower_leg')) |
|  | rs17854357 | ENSG00000172803.13 | [SNX32](javascript:portalClient.eqtl.gotoGeneExpression('SNX32')) | 4.90E-16 | 1.3 | [Cells - EBV-transformed lymphocytes](javascript:portalClient.eqtl.goTissuePage('Cells_EBV-transformed_lymphocytes')) |
|  | rs17854357 | ENSG00000172543.3 | [CTSW](javascript:portalClient.eqtl.gotoGeneExpression('CTSW')) | 6.90E-16 | -0.77 | [Artery - Aorta](javascript:portalClient.eqtl.goTissuePage('Artery_Aorta')) |
|  | rs17854357 | ENSG00000172803.13 | [SNX32](javascript:portalClient.eqtl.gotoGeneExpression('SNX32')) | 1.40E-15 | 1.1 | [Esophagus - Gastroesophageal Junction](javascript:portalClient.eqtl.goTissuePage('Esophagus_Gastroesophageal_Junction')) |
|  | rs17854357 | ENSG00000172803.13 | [SNX32](javascript:portalClient.eqtl.gotoGeneExpression('SNX32')) | 1.80E-15 | 1.2 | [Artery - Coronary](javascript:portalClient.eqtl.goTissuePage('Artery_Coronary')) |
|  | rs17854357 | ENSG00000172803.13 | [SNX32](javascript:portalClient.eqtl.gotoGeneExpression('SNX32')) | 4.00E-15 | 0.97 | [Heart - Left Ventricle](javascript:portalClient.eqtl.goTissuePage('Heart_Left_Ventricle')) |
|  | rs17854357 | ENSG00000172543.3 | [CTSW](javascript:portalClient.eqtl.gotoGeneExpression('CTSW')) | 4.70E-15 | -0.6 | [Lung](javascript:portalClient.eqtl.goTissuePage('Lung')) |
|  | rs17854357 | ENSG00000172803.13 | [SNX32](javascript:portalClient.eqtl.gotoGeneExpression('SNX32')) | 1.30E-14 | 0.77 | [Whole Blood](javascript:portalClient.eqtl.goTissuePage('Whole_Blood')) |
|  | rs17854357 | ENSG00000172500.8 | [FIBP](javascript:portalClient.eqtl.gotoGeneExpression('FIBP')) | 1.40E-14 | 0.33 | [Muscle - Skeletal](javascript:portalClient.eqtl.goTissuePage('Muscle_Skeletal')) |
|  | rs17854357 | ENSG00000172543.3 | [CTSW](javascript:portalClient.eqtl.gotoGeneExpression('CTSW')) | 4.40E-14 | -0.62 | [Adipose - Subcutaneous](javascript:portalClient.eqtl.goTissuePage('Adipose_Subcutaneous')) |
|  | rs17854357 | ENSG00000172543.3 | [CTSW](javascript:portalClient.eqtl.gotoGeneExpression('CTSW')) | 8.80E-14 | -0.77 | [Stomach](javascript:portalClient.eqtl.goTissuePage('Stomach')) |
|  | rs17854357 | ENSG00000172803.13 | [SNX32](javascript:portalClient.eqtl.gotoGeneExpression('SNX32')) | 1.20E-13 | 0.98 | [Pancreas](javascript:portalClient.eqtl.goTissuePage('Pancreas')) |
|  | rs17854357 | ENSG00000172543.3 | [CTSW](javascript:portalClient.eqtl.gotoGeneExpression('CTSW')) | 1.80E-13 | -0.62 | [Adipose - Visceral (Omentum)](javascript:portalClient.eqtl.goTissuePage('Adipose_Visceral_Omentum')) |
|  | rs17854357 | ENSG00000172803.13 | [SNX32](javascript:portalClient.eqtl.gotoGeneExpression('SNX32')) | 3.90E-13 | 1.1 | [Small Intestine - Terminal Ileum](javascript:portalClient.eqtl.goTissuePage('Small_Intestine_Terminal_Ileum')) |
|  | rs17854357 | ENSG00000172543.3 | [CTSW](javascript:portalClient.eqtl.gotoGeneExpression('CTSW')) | 6.20E-13 | -0.58 | [Artery - Tibial](javascript:portalClient.eqtl.goTissuePage('Artery_Tibial')) |
|  | rs17854357 | ENSG00000172543.3 | [CTSW](javascript:portalClient.eqtl.gotoGeneExpression('CTSW')) | 6.50E-13 | -0.27 | [Whole Blood](javascript:portalClient.eqtl.goTissuePage('Whole_Blood')) |
|  | rs17854357 | ENSG00000172543.3 | [CTSW](javascript:portalClient.eqtl.gotoGeneExpression('CTSW')) | 1.40E-12 | -0.72 | [Breast - Mammary Tissue](javascript:portalClient.eqtl.goTissuePage('Breast_Mammary_Tissue')) |
|  | rs17854357 | ENSG00000172500.8 | [FIBP](javascript:portalClient.eqtl.gotoGeneExpression('FIBP')) | 4.90E-12 | 0.38 | [Cells - Transformed fibroblasts](javascript:portalClient.eqtl.goTissuePage('Cells_Transformed_fibroblasts')) |
|  | rs17854357 | ENSG00000172803.13 | [SNX32](javascript:portalClient.eqtl.gotoGeneExpression('SNX32')) | 1.30E-11 | 1.5 | [Pituitary](javascript:portalClient.eqtl.goTissuePage('Pituitary')) |
|  | rs17854357 | ENSG00000172803.13 | [SNX32](javascript:portalClient.eqtl.gotoGeneExpression('SNX32')) | 1.00E-10 | 0.84 | [Adrenal Gland](javascript:portalClient.eqtl.goTissuePage('Adrenal_Gland')) |
|  | rs17854357 | ENSG00000172803.13 | [SNX32](javascript:portalClient.eqtl.gotoGeneExpression('SNX32')) | 1.10E-10 | 0.84 | [Brain - Anterior cingulate cortex (BA24)](javascript:portalClient.eqtl.goTissuePage('Brain_Anterior_cingulate_cortex_BA24')) |
|  | rs17854357 | ENSG00000172803.13 | [SNX32](javascript:portalClient.eqtl.gotoGeneExpression('SNX32')) | 1.20E-10 | 0.89 | [Brain - Frontal Cortex (BA9)](javascript:portalClient.eqtl.goTissuePage('Brain_Frontal_Cortex_BA9')) |
|  | rs17854357 | ENSG00000172803.13 | [SNX32](javascript:portalClient.eqtl.gotoGeneExpression('SNX32')) | 1.90E-10 | 0.84 | [Ovary](javascript:portalClient.eqtl.goTissuePage('Ovary')) |
|  | rs17854357 | ENSG00000172803.13 | [SNX32](javascript:portalClient.eqtl.gotoGeneExpression('SNX32')) | 3.90E-10 | 0.94 | [Vagina](javascript:portalClient.eqtl.goTissuePage('Vagina')) |
|  | rs17854357 | ENSG00000172500.8 | [FIBP](javascript:portalClient.eqtl.gotoGeneExpression('FIBP')) | 6.30E-10 | 0.56 | [Cells - EBV-transformed lymphocytes](javascript:portalClient.eqtl.goTissuePage('Cells_EBV-transformed_lymphocytes')) |
|  | rs17854357 | ENSG00000172803.13 | [SNX32](javascript:portalClient.eqtl.gotoGeneExpression('SNX32')) | 7.70E-10 | 1 | [Brain - Hypothalamus](javascript:portalClient.eqtl.goTissuePage('Brain_Hypothalamus')) |
|  | rs17854357 | ENSG00000172543.3 | [CTSW](javascript:portalClient.eqtl.gotoGeneExpression('CTSW')) | 1.30E-09 | -0.85 | [Small Intestine - Terminal Ileum](javascript:portalClient.eqtl.goTissuePage('Small_Intestine_Terminal_Ileum')) |
|  | rs17854357 | ENSG00000172543.3 | [CTSW](javascript:portalClient.eqtl.gotoGeneExpression('CTSW')) | 1.90E-09 | -0.57 | [Skin - Not Sun Exposed (Suprapubic)](javascript:portalClient.eqtl.goTissuePage('Skin_Not_Sun_Exposed_Suprapubic')) |
|  | rs17854357 | ENSG00000172803.13 | [SNX32](javascript:portalClient.eqtl.gotoGeneExpression('SNX32')) | 3.80E-09 | 0.63 | [Testis](javascript:portalClient.eqtl.goTissuePage('Testis')) |
|  | rs17854357 | ENSG00000172803.13 | [SNX32](javascript:portalClient.eqtl.gotoGeneExpression('SNX32')) | 4.20E-09 | 0.54 | [Muscle - Skeletal](javascript:portalClient.eqtl.goTissuePage('Muscle_Skeletal')) |
|  | rs17854357 | ENSG00000172500.8 | [FIBP](javascript:portalClient.eqtl.gotoGeneExpression('FIBP')) | 4.90E-09 | 0.45 | [Heart - Left Ventricle](javascript:portalClient.eqtl.goTissuePage('Heart_Left_Ventricle')) |
|  | rs17854357 | ENSG00000172543.3 | [CTSW](javascript:portalClient.eqtl.gotoGeneExpression('CTSW')) | 1.10E-08 | -0.71 | [Pancreas](javascript:portalClient.eqtl.goTissuePage('Pancreas')) |
|  | rs17854357 | ENSG00000172500.8 | [FIBP](javascript:portalClient.eqtl.gotoGeneExpression('FIBP')) | 5.50E-08 | 0.53 | [Testis](javascript:portalClient.eqtl.goTissuePage('Testis')) |
|  | rs17854357 | ENSG00000172543.3 | [CTSW](javascript:portalClient.eqtl.gotoGeneExpression('CTSW')) | 5.60E-08 | -0.75 | [Adrenal Gland](javascript:portalClient.eqtl.goTissuePage('Adrenal_Gland')) |
|  | rs17854357 | ENSG00000172543.3 | [CTSW](javascript:portalClient.eqtl.gotoGeneExpression('CTSW')) | 1.10E-07 | -0.77 | [Esophagus - Gastroesophageal Junction](javascript:portalClient.eqtl.goTissuePage('Esophagus_Gastroesophageal_Junction')) |
|  | rs17854357 | ENSG00000172500.8 | [FIBP](javascript:portalClient.eqtl.gotoGeneExpression('FIBP')) | 2.80E-07 | 0.42 | [Heart - Atrial Appendage](javascript:portalClient.eqtl.goTissuePage('Heart_Atrial_Appendage')) |
|  | rs17854357 | ENSG00000172543.3 | [CTSW](javascript:portalClient.eqtl.gotoGeneExpression('CTSW')) | 4.30E-07 | -0.88 | [Spleen](javascript:portalClient.eqtl.goTissuePage('Spleen')) |
|  | rs17854357 | ENSG00000172543.3 | [CTSW](javascript:portalClient.eqtl.gotoGeneExpression('CTSW')) | 6.90E-07 | -0.79 | [Vagina](javascript:portalClient.eqtl.goTissuePage('Vagina')) |
|  | rs17854357 | ENSG00000172543.3 | [CTSW](javascript:portalClient.eqtl.gotoGeneExpression('CTSW')) | 7.00E-07 | -1 | [Prostate](javascript:portalClient.eqtl.goTissuePage('Prostate')) |
|  | rs17854357 | ENSG00000172803.13 | [SNX32](javascript:portalClient.eqtl.gotoGeneExpression('SNX32')) | 0.0000011 | 1 | [Prostate](javascript:portalClient.eqtl.goTissuePage('Prostate')) |
|  | rs17854357 | ENSG00000172803.13 | [SNX32](javascript:portalClient.eqtl.gotoGeneExpression('SNX32')) | 0.0000035 | 0.89 | [Spleen](javascript:portalClient.eqtl.goTissuePage('Spleen')) |
|  | rs17854357 | ENSG00000172500.8 | [FIBP](javascript:portalClient.eqtl.gotoGeneExpression('FIBP')) | 0.0000045 | 0.22 | [Esophagus - Muscularis](javascript:portalClient.eqtl.goTissuePage('Esophagus_Muscularis')) |
|  | rs17854357 | ENSG00000172543.3 | [CTSW](javascript:portalClient.eqtl.gotoGeneExpression('CTSW')) | 0.000006 | -0.45 | [Cells - Transformed fibroblasts](javascript:portalClient.eqtl.goTissuePage('Cells_Transformed_fibroblasts')) |
|  | rs17854357 | ENSG00000172500.8 | [FIBP](javascript:portalClient.eqtl.gotoGeneExpression('FIBP')) | 0.0000065 | 0.16 | [Artery - Tibial](javascript:portalClient.eqtl.goTissuePage('Artery_Tibial')) |
|  | rs17854357 | ENSG00000172500.8 | [FIBP](javascript:portalClient.eqtl.gotoGeneExpression('FIBP')) | 0.0000095 | 0.22 | [Nerve - Tibial](javascript:portalClient.eqtl.goTissuePage('Nerve_Tibial')) |
|  | rs17854357 | ENSG00000172543.3 | [CTSW](javascript:portalClient.eqtl.gotoGeneExpression('CTSW')) | 0.000018 | -0.36 | [Muscle - Skeletal](javascript:portalClient.eqtl.goTissuePage('Muscle_Skeletal')) |
|  | rs17854357 | ENSG00000172500.8 | [FIBP](javascript:portalClient.eqtl.gotoGeneExpression('FIBP')) | 0.000022 | 0.22 | [Adipose - Subcutaneous](javascript:portalClient.eqtl.goTissuePage('Adipose_Subcutaneous')) |
| 16q24.3 | rs1805007 | ENSG00000003249.9 | DBNDD1 | 4.50E-16 | 0.68 | Thyroid |
|  | rs1805007 | ENSG00000003249.9 | DBNDD1 | 2.20E-14 | 0.94 | Artery - Tibial |
|  | rs1805007 | ENSG00000003249.9 | DBNDD1 | 3.70E-14 | 0.97 | Nerve - Tibial |
|  | rs1805007 | ENSG00000003249.9 | DBNDD1 | 9.20E-14 | 1.3 | Heart - Atrial Appendage |
|  | rs1805007 | ENSG00000003249.9 | DBNDD1 | 1.60E-13 | 0.75 | Lung |
|  | rs1805007 | ENSG00000003249.9 | DBNDD1 | 3.00E-13 | 1.3 | Colon - Transverse |
|  | rs1805007 | ENSG00000221819.2 | C16orf3 | 1.20E-12 | 1.7 | Brain - Cerebellum |
|  | rs1805007 | ENSG00000141013.10 | GAS8 | 1.20E-12 | 1.5 | Brain - Cerebellum |
|  | rs1805007 | ENSG00000222019.3 | URAHP | 4.10E-12 | 1.3 | Brain - Cerebellum |
|  | rs1805007 | ENSG00000003249.9 | DBNDD1 | 4.20E-10 | 0.73 | Heart - Left Ventricle |
|  | rs1805007 | ENSG00000003249.9 | DBNDD1 | 5.60E-10 | 0.82 | Skin - Not Sun Exposed (Suprapubic) |
|  | rs1805007 | ENSG00000003249.9 | DBNDD1 | 7.80E-10 | 0.86 | Cells - Transformed fibroblasts |
|  | rs1805007 | ENSG00000222019.3 | URAHP | 7.80E-10 | 1.2 | Brain - Cerebellar Hemisphere |
|  | rs1805007 | ENSG00000141013.10 | GAS8 | 4.20E-09 | 1.3 | Brain - Cerebellar Hemisphere |
|  | rs1805007 | ENSG00000221819.2 | C16orf3 | 3.50E-08 | 1.2 | Brain - Cerebellar Hemisphere |
|  | rs1805007 | ENSG00000003249.9 | DBNDD1 | 5.20E-08 | 0.91 | Adrenal Gland |
|  | rs1805007 | ENSG00000141013.10 | GAS8 | 5.40E-08 | 1.1 | Brain - Putamen (basal ganglia) |
|  | rs1805007 | ENSG00000222019.3 | URAHP | 6.10E-08 | 0.99 | Brain - Cortex |
|  | rs1805007 | ENSG00000221819.2 | C16orf3 | 7.70E-08 | 1.2 | Brain - Cortex |
|  | rs1805007 | ENSG00000003249.9 | DBNDD1 | 8.50E-08 | 0.63 | Pancreas |
|  | rs1805007 | ENSG00000003249.9 | DBNDD1 | 1.00E-07 | 0.54 | Skin - Sun Exposed (Lower leg) |
|  | rs1805007 | ENSG00000003249.9 | DBNDD1 | 2.00E-07 | 0.88 | Colon - Sigmoid |
|  | rs1805007 | ENSG00000003249.9 | DBNDD1 | 2.50E-07 | 0.46 | Esophagus - Muscularis |
|  | rs1805007 | ENSG00000003249.9 | DBNDD1 | 3.40E-07 | 0.55 | Stomach |
|  | rs1805007 | ENSG00000003249.9 | DBNDD1 | 7.60E-07 | 0.63 | Adipose - Subcutaneous |
|  | rs1805007 | ENSG00000141013.10 | GAS8 | 0.0000012 | 0.95 | Brain - Cortex |
|  | rs1805007 | ENSG00000141013.10 | GAS8 | 0.0000022 | -0.51 | Muscle - Skeletal |
|  | rs1805007 | ENSG00000167523.9 | SPATA33 | 0.0000029 | -0.56 | Skin - Sun Exposed (Lower leg) |
|  | rs1805007 | ENSG00000222019.3 | URAHP | 0.0000067 | -0.47 | Muscle - Skeletal |
|  | rs1805007 | ENSG00000187741.10 | FANCA | 0.0000067 | -0.25 | Cells - Transformed fibroblasts |
|  | rs1805007 | ENSG00000167523.9 | SPATA33 | 0.000014 | -0.48 | Thyroid |
| Variants identified for each region were evaluated to determine if they were *cis* eQTLs using publically available data in GTEx (13). For a given SNP, the *p* value and effect size are provided for each significant gene (Genecode ID/Gene Symbol) and tissue pair. | | | | | | |

**Supplementary Table 4. Cross-tabulation comparison of "rosacea symptom severity" cohort and the "rosacea diagnosis" primary cohort published in Chang et al. 2015 [9]**

| Rosacea Symptom Severity Score Group | Rosacea symptom_severity cohort | Rosacea diagnosis (cases/ controls) from Chang et al. 2015_primary cohort | | Participants in the "rosacea_severity" cohort not included in the "rosacea_diagnosis" primary cohort |
| --- | --- | --- | --- | --- |
|  |  | no (controls) | yes (cases) |  |
| [0-1] | 34393 | 10095 | 119 | 24179 |
| (1-2] | 9431 | 2485 | 87 | 6859 |
| (2-3] | 7494 | 1751 | 157 | 5586 |
| (3-5] | 9124 | 2004 | 365 | 6755 |
| (5-8] | 6533 | 1197 | 498 | 4838 |
| (8-32] | 6290 | 733 | 1006 | 4551 |

**Supplementary Figure 1. Regional association plots for GWAS association variants.** The regional association plots show association test statistics versus position in the vicinity of the strongest associations. The *y*-axis shows negative transformed –log_10_ of association test *p* values, and *x*-axis shows the adjacent region on the chromosome. The plots are generated in R, using linkage disequilibrium statistics (R^2^) calculated from the phase 1 release of the 1000 Genomic data (European population). In the plot, a triangle point indicates SNP in the PICS set, and a circle indicates genotyped or imputed SNP. Red color indicates strength of linkage disequilibrium with the index SNP (blue triangle).

Supplemental Figure 1a: Regional association plot for rs12203592

******

Supplemental Figure 1b: Regional association plot for rs1129038****

Supplemental Figure 1c: Regional association plot for rs16891982****

Supplemental Figure 1d: Regional association plot for rs847

Supplemental Figure 1e: Regional association plot for rs149851565

Supplemental Figure 1f: Regional association plot for rs77779142

Supplemental Figure 1g: Regional association plot for rs1805007******

**Supplementary Figure 2. Regulatory chromatin marks present in immune and blood cell lines at the IRF4 locus**

Regulatory elements are marked by active histone marks (H3K27ac, H3K9ac, H3K4me3), open chromatin marks (DNase), and transcription factor binding sites (TF ChIP-seq). Represented skin cell lines include dermal fibroblast (NHDF), fetal skin (FSK), fetal abdominal skin (FFSA), and forekin melanocytes (PFM). Represented immune cell lines include B-lymphocytes (GM12878), CD8 primary cells, CD8 memory cells, CD4 primary cells, and CD4 memory cells. Blue highlighted region represents a regulatory element in at least one cell line that contains a GWAS SNP.


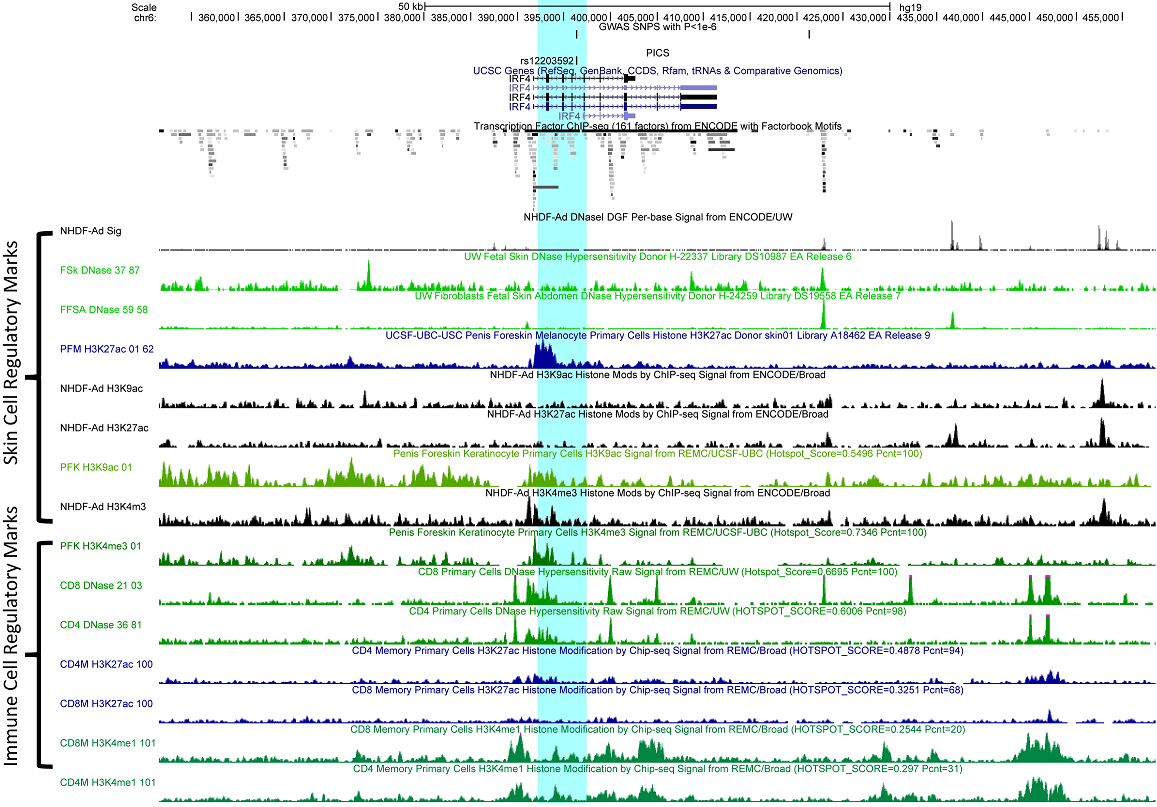


**Supplementary Figure 3. Regulatory chromatin marks present in immune and blood cell lines at the HERC2 locus**

Regulatory elements are marked by active histone marks (H3K27ac, H3K9ac, H3K4me3), open chromatin marks (DNase), and transcription factor binding sites (TF ChIP-seq). Represented skin cell lines include dermal fibroblast (NHDF), fetal skin (FSK), fetal abdominal skin (FFSA), and forekin melanocytes (PFM). Represented immune cell lines include B-lymphocytes (GM12878), CD8 primary cells, CD8 memory cells, CD4 primary cells, and CD4 memory cells. Blue highlighted region represents a regulatory element in at least one cell line that contains a GWAS SNP.


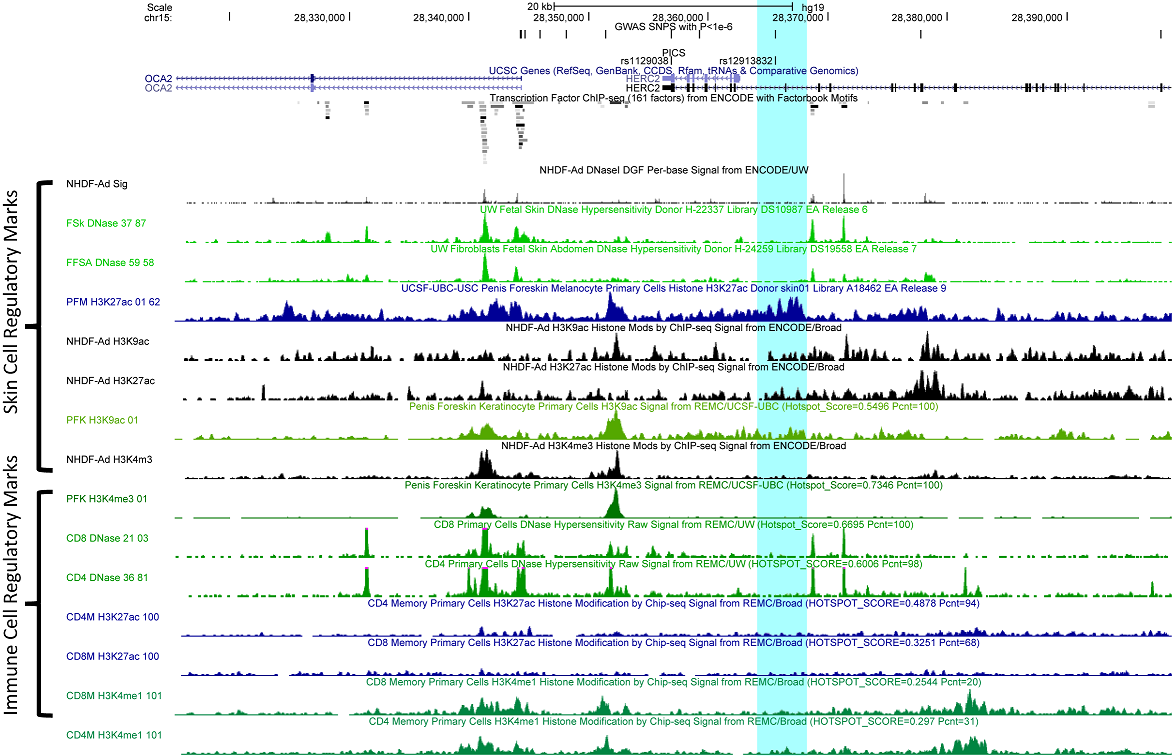


**Supplementary Figure 4. Regulatory chromatin marks present in immune and blood cell lines at the SLC45A2 locus**

Regulatory elements are marked by active histone marks (H3K27ac, H3K9ac, H3K4me3), open chromatin marks (DNase), and transcription factor binding sites (TF ChIP-seq). Represented skin cell lines include dermal fibroblast (NHDF), fetal skin (FSK), fetal abdominal skin (FFSA), and forekin melanocytes (PFM). Represented immune cell lines include B-lymphocytes (GM12878), CD8 primary cells, CD8 memory cells, CD4 primary cells, and CD4 memory cells.


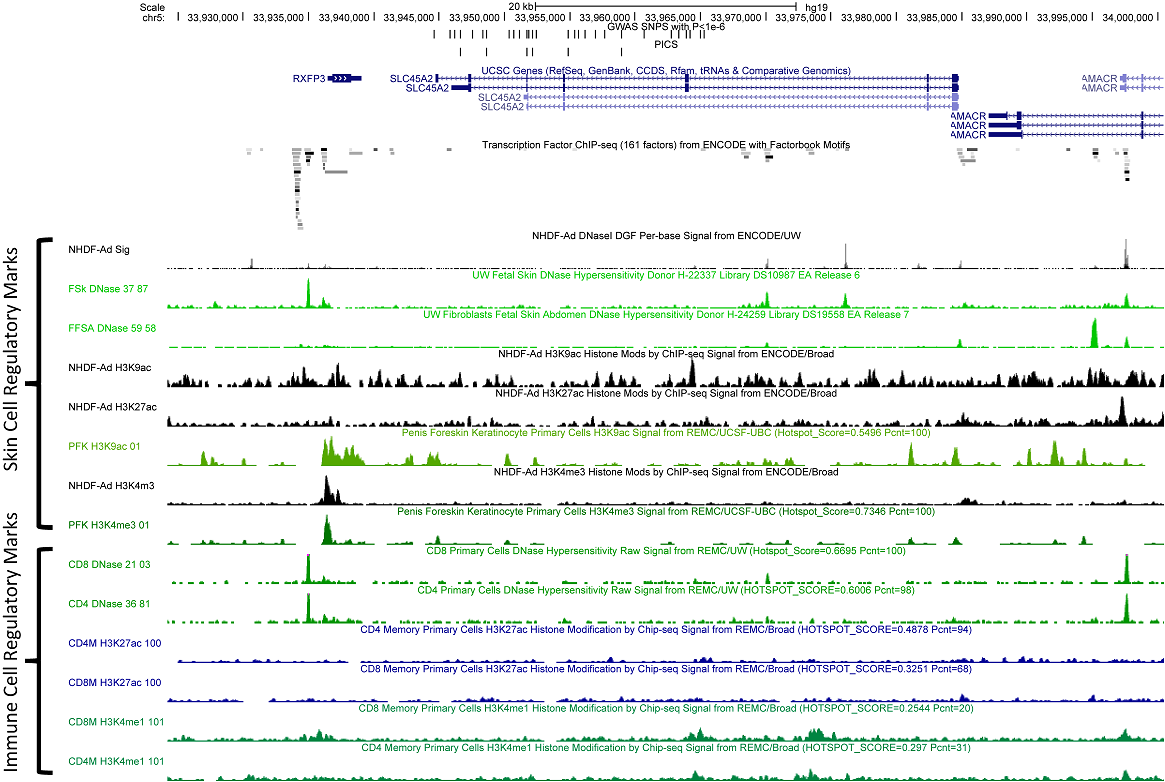


**Supplementary Figure 5. Regulatory chromatin marks present in immune and blood cell lines at the IL13 locus**

Regulatory elements are marked by active histone marks (H3K27ac, H3K9ac, H3K4me3), open chromatin marks (DNase), and transcription factor binding sites (TF ChIP-seq). Represented skin cell lines include dermal fibroblast (NHDF), fetal skin (FSK), fetal abdominal skin (FFSA), and forekin melanocytes (PFM). Represented immune cell lines include B-lymphocytes (GM12878), CD8 primary cells, CD8 memory cells, CD4 primary cells, and CD4 memory cells. Blue highlighted region represents a regulatory element in at least one cell line that contains a GWAS SNP.


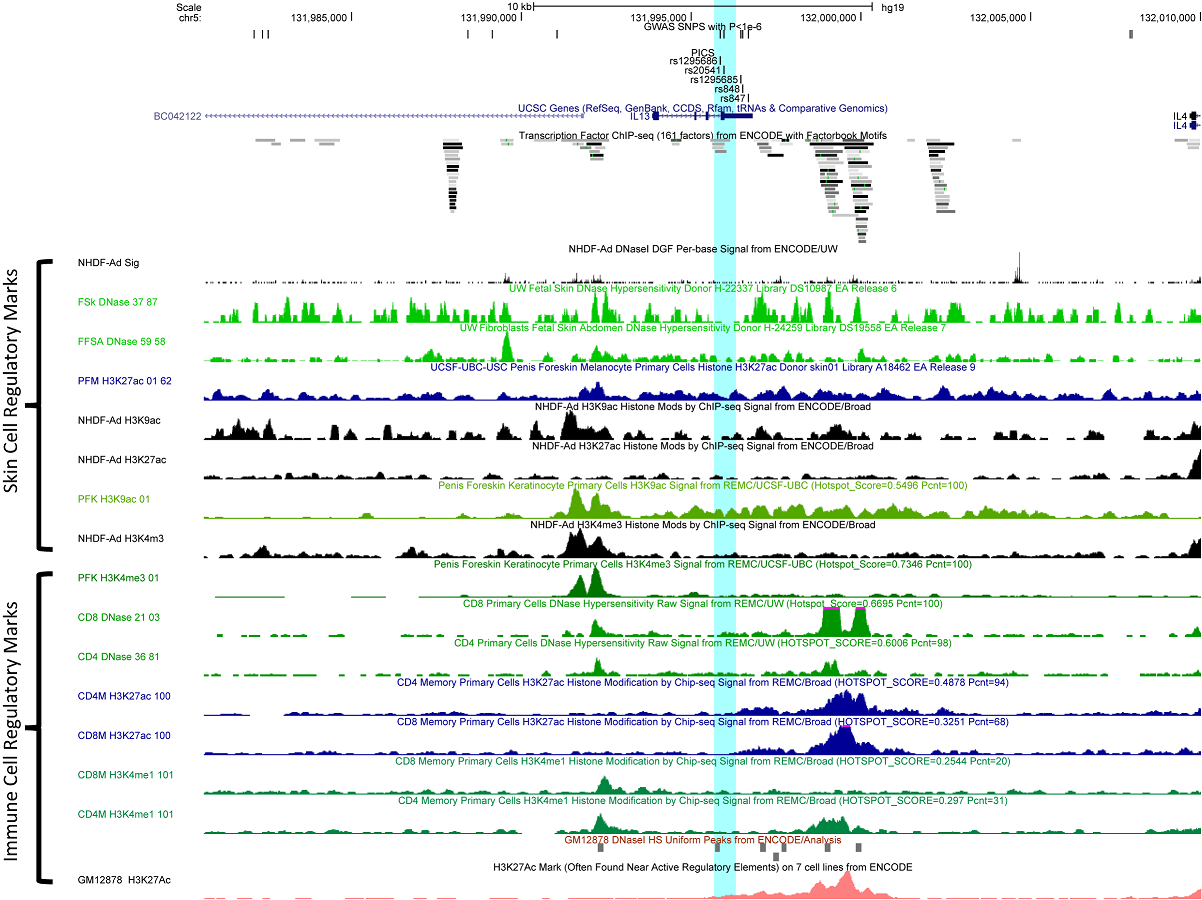


**Supplementary Figure 6. Regulatory chromatin marks present in immune and blood cell lines at the NRXN3 locus**

Regulatory elements are marked by active histone marks (H3K27ac, H3K9ac, H3K4me3), open chromatin marks (DNase), and transcription factor binding sites (TF ChIP-seq). Represented skin cell lines include dermal fibroblast (NHDF), fetal skin (FSK), fetal abdominal skin (FFSA), and forekin melanocytes (PFM). Represented immune cell lines include B-lymphocytes (GM12878), CD8 primary cells, CD8 memory cells, CD4 primary cells, and CD4 memory cells.


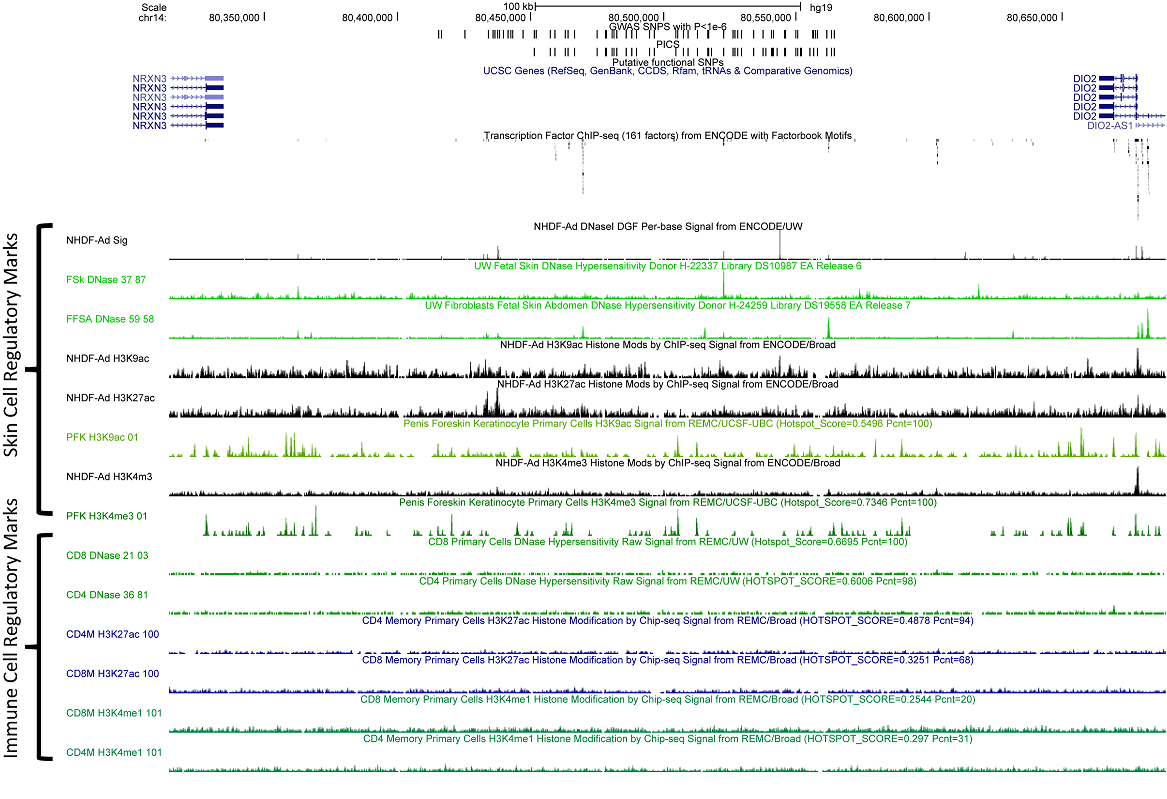


**Supplementary Figure 7. Regulatory chromatin marks present in immune and blood cell lines at the SNX32 locus**

Regulatory elements are marked by active histone marks (H3K27ac, H3K9ac, H3K4me3), open chromatin marks (DNase), and transcription factor binding sites (TF ChIP-seq). Represented skin cell lines include dermal fibroblast (NHDF), fetal skin (FSK), fetal abdominal skin (FFSA), and forekin melanocytes (PFM). Represented immune cell lines include B-lymphocytes (GM12878), CD8 primary cells, CD8 memory cells, CD4 primary cells, and CD4 memory cells. Blue highlighted region represents a regulatory element in at least one cell line that contains a GWAS SNP.


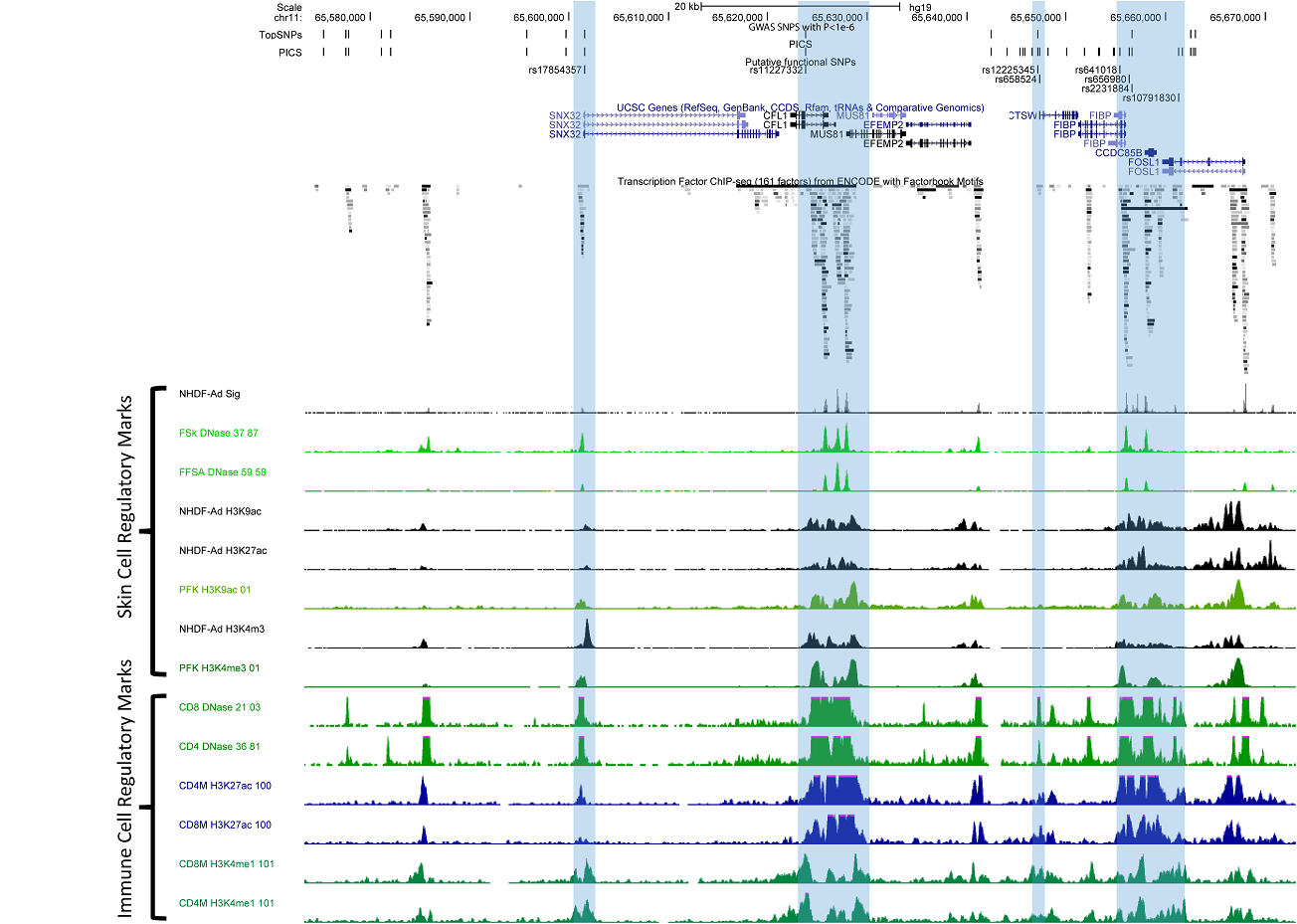


**Supplementary Figure 8. Q-Q Plot of GWAS Results**

The Quantile-quantile (Q-Q) plot depicts observed versus expected quantiles for the GWAS *p* values, where the expected distribution of *p* values is uniform under the null hypothesis, plotted on a log scale. A solid red line is shown with a slope of 1, and dashed red lines represent a 95% confidence envelope under the assumption that the test results are independent.


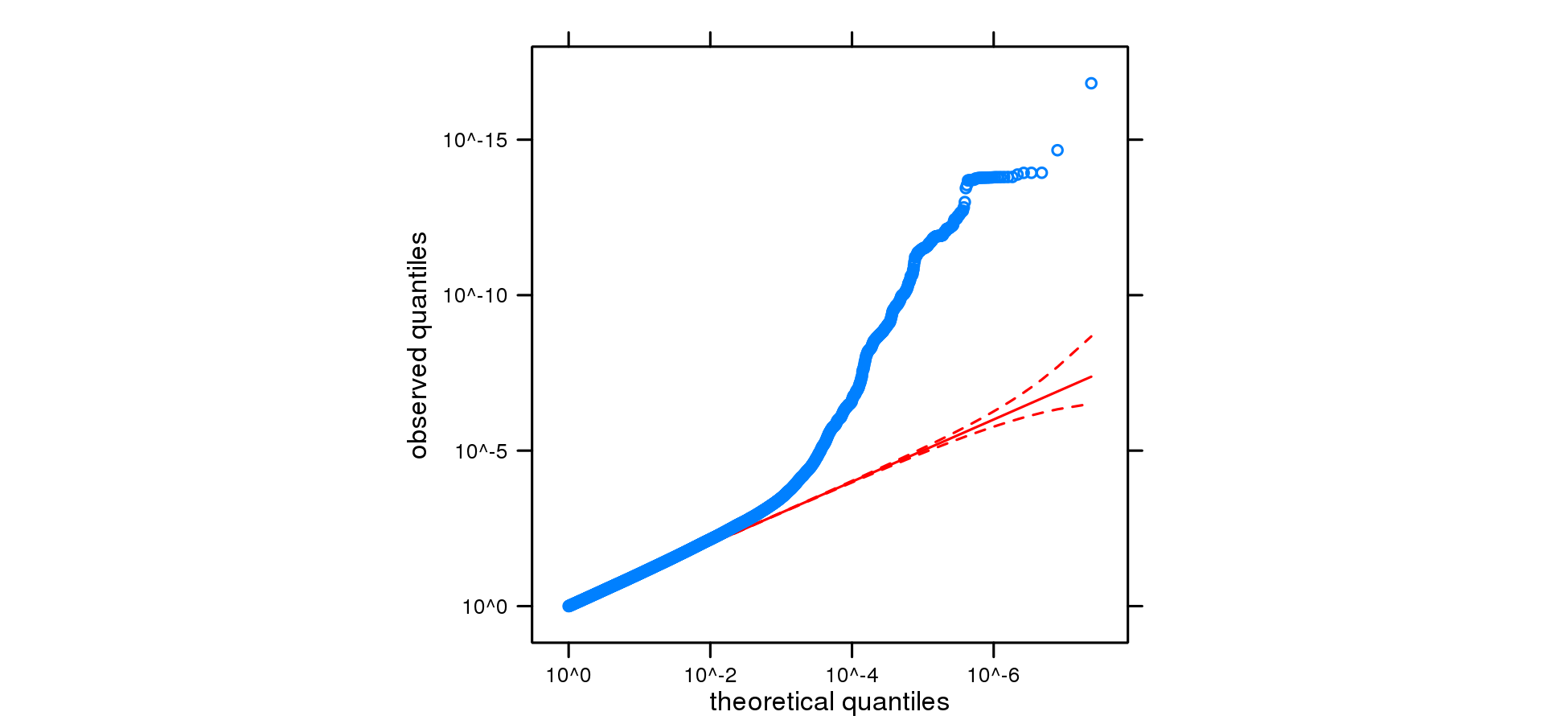

Supplement: ddy184_supplmaterials_clean_v3 [file ddy184_supplmaterials_clean_v3.docx]
